# Imaging of Dysfunctional Elastogenesis in Atherosclerosis Using an Improved Gadolinium-Based Tetrameric MRI Probe Targeted to Tropoelastin

**DOI:** 10.1021/acs.jmedchem.1c01286

**Published:** 2021-10-18

**Authors:** Federico Capuana, Alkystis Phinikaridou, Rachele Stefania, Sergio Padovan, Begoña Lavin, Sara Lacerda, Eyad Almouazen, Yves Chevalier, Laurence Heinrich-Balard, René M. Botnar, Silvio Aime, Giuseppe Digilio

**Affiliations:** † Department of Molecular Biotechnology and Health Sciences, University of Turin, Via Nizza 52, Turin 10126, Italy; ‡ School of Biomedical Engineering and Imaging Sciences, King’s College London, Westminster Bridge Road, London SE1 7EH, United Kingdom; ∥ Institute for Biostructures and Bioimages (CNR) c/o Molecular Biotechnology Center, Via Nizza 52, Torino 10126, Italy; ⊥ Department of Biochemistry and Molecular Biology, School of Chemistry, Complutense University, Ciudad Universitaria s/n, Madrid 28040, Spain; # Centre de Biophysique Moléculaire, CNRS, UPR 4301, Université d’Orléans, Rue Charles Sadron, Orléans Cedex 2 45071, France; ∇ CNRS, LAGEPP UMR 5007, Univ Lyon, Université Claude Bernard Lyon 1, 43 boulevard du 11 novembre 1918, Villeurbanne 69622, France; ○ INSA Lyon, CNRS, MATEIS, UMR5510, Univ Lyon, Université Claude Bernard Lyon 1, Villeurbanne 69100, France; ◆ Escuela de Ingeniería, Pontificia Universidad Católica de Chile, Avda. Vicuña Mackenna, Santiago 4860, Chile; ¶ IRCCS SDN, Napoli 80100, Italy; & Department of Science and Technologic Innovation, Università del Piemonte Orientale ″Amedeo Avogadro″, Viale T. Michel 11, Alessandria 15121, Italy

## Abstract

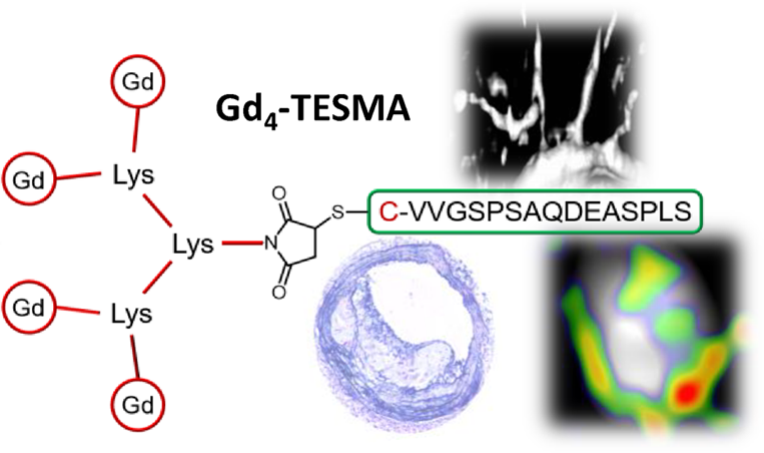

Dysfunctional elastin turnover plays a major role in the progression of atherosclerotic plaques. Failure of tropoelastin cross-linking into mature elastin leads to the accumulation of tropoelastin within the growing plaque, increasing its instability. Here we present Gd_4_-TESMA, an MRI contrast agent specifically designed for molecular imaging of tropoelastin within plaques. Gd_4_-TESMA is a tetrameric probe composed of a tropoelastin-binding peptide (the VVGS-peptide) conjugated with four Gd(III)-DOTA-monoamide chelates. It shows a relaxivity per molecule of 34.0 ± 0.8 mM^–1^ s^–1^ (20 MHz, 298 K, pH 7.2), a good binding affinity to tropoelastin (*K*_D_ = 41 ± 12 μM), and a serum half-life longer than 2 h. Gd_4_-TESMA accumulates specifically in atherosclerotic plaques in the ApoE^–/–^ murine model of plaque progression, with 2 h persistence of contrast enhancement. As compared to the monomeric counterpart (Gd-TESMA), the tetrameric Gd_4_-TESMA probe shows a clear advantage regarding both sensitivity and imaging time window, allowing for a better characterization of atherosclerotic plaques.

## Introduction

Atherosclerotic plaque rupture is one of the leading causes of life-threatening cardiovascular events. The size of atherosclerotic plaques and the extent of luminal stenosis can be assessed by several imaging techniques, but these morphological parameters alone are not reliable diagnostic indicators of the risk for plaque rupture.^1^ Depending on the morphology, composition, and histopathologic features, plaques have been classified as stable or high risk (vulnerable/unstable). Pathology studies revealed that plaques at high risk of rupturing with ensuing thrombosis are characterized by a thin fibrous cap, a large necrotic lipid-rich core, microcalcifications, and increased macrophage infiltration.^2^ Increased plaque microvessel density may lead to intraplaque hemorrhage, further increasing the risk of rupture.^3^ Phenotyping of atherosclerotic plaques by noninvasive imaging techniques has emerged as an essential requirement for risk assessment.^4^ Vulnerable-plaque imaging aims at detecting biomarkers that would allow classifying plaques according to their risk of rupture.

Elastin is a major component of the arterial wall matrix. Dysfunctional elastin turnover promotes atherosclerotic plaque progression.^5−10^ This turnover involves a balance between elastolysis—the degradation of fibers and elastogenesis—*de novo* synthesis, and cross-linking of the newly synthesized soluble tropoelastin (TE) precursor into mature polymeric elastin.^11^ In atherosclerosis, elastolysis is enhanced by inflammation-driven upregulation of elastases.^12^ Simultaneously, elastin synthesis resumes and new tropoelastin molecules are produced by plaque macrophages and vascular smooth muscle cells.^5^ However, tropoelastin monomers frequently fail to cross-link into mature polymeric elastin as a result of the reduced expression or absence of lysyl oxidase (LOX)^13−15^ or any of the components of the microfibrillar scaffold required for fiber assembly.^16−19^ Therefore, tropoelastin accumulates within the growing lesion, promoting plaque progression and instability.^9^

**Scheme 1:**
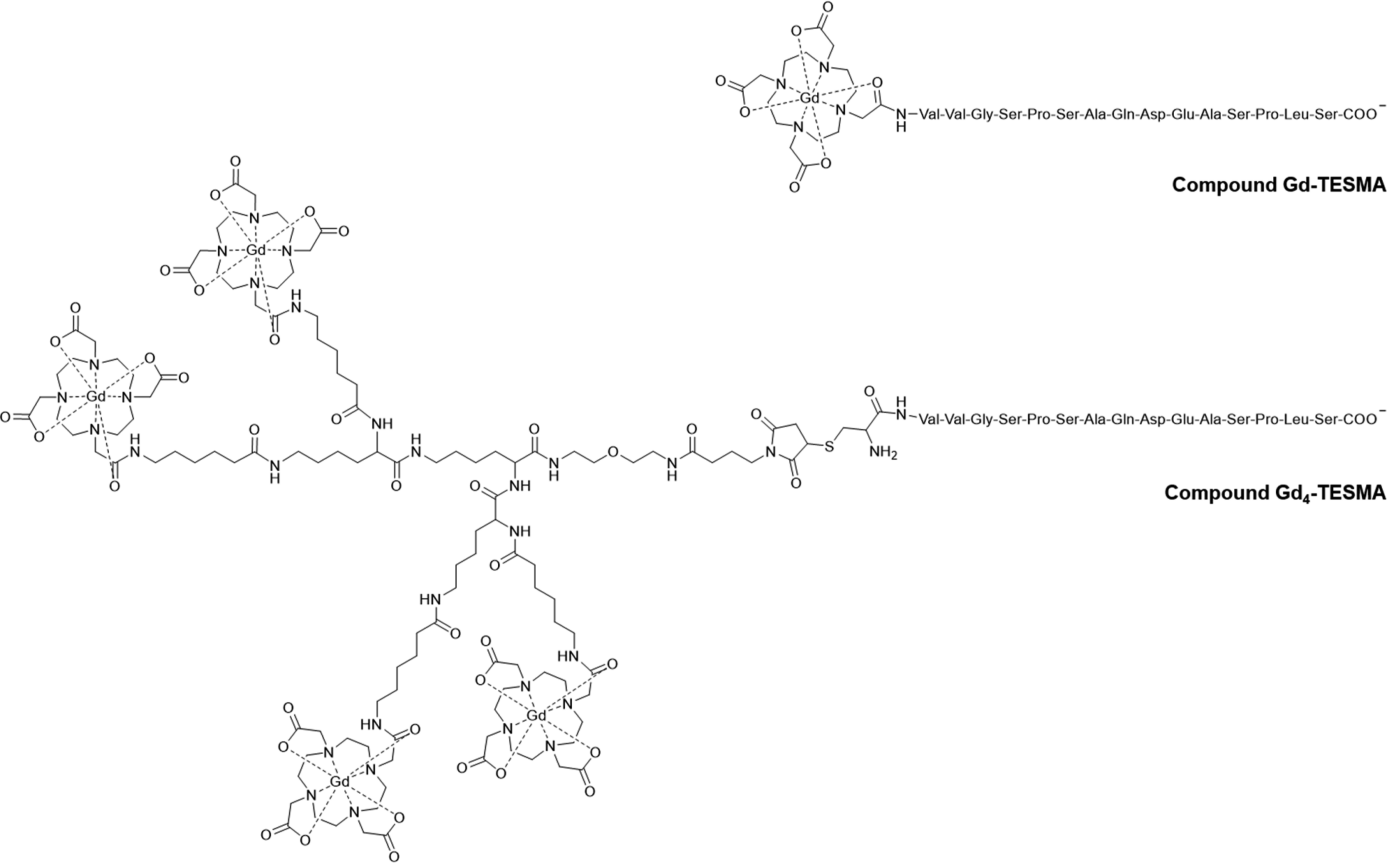
Chemical Structures of Gd-TESMA and Gd_4_-TESMA

Imaging probes targeting plaque elastin and tropoelastin have been developed to enable the quantitative assessment of elastin turnover and the phenotyping of plaques and to assess their potential for rupture. Most of the studies in preclinical models of atherosclerosis^10,20,21^ and other vascular diseases^22−25^ focused on total elastin imaging by using a low molar mass probe (Gd-ESMA).^24,26^ This elastin-specific MR agent binds to both mature/cross-linked and monomeric tropoelastin molecules. More recently, a new probe designed to specifically bind to monomeric tropoelastin has been used for plaque phenotyping.^9,27^ This molecular probe, called Gd-TESMA (Scheme 1), consists of a single Gd-DOTA monoamide unit (DOTA = 1,4,7,10-tetraazacyclododecane-1,4,7,10-tetraacetic acid) conjugated with a 15-residue peptide sequence (named VVGS-peptide) that showed a good binding affinity for tropoelastin and good selectivity over elastin and a panel of other matrix proteins.

Studies in an apolipoprotein-E-deficient (ApoE^–/–^) murine model of progressive atherosclerosis and abdominal aortic aneurysm showed improved visualization of vessel walls during disease progression that correlated with the levels of tropoelastin as detected by *ex vivo* histology and Western blotting. Interestingly, studies in a rabbit model of plaque rupture showed higher tropoelastin content and Gd-induced relaxation rates in rupture-prone compared with stable plaques. Therefore, tropoelastin is a novel MRI biomarker for atherosclerotic plaque progression and instability, and aortic aneurysm expansion.^9,27^

**Figure 1:**
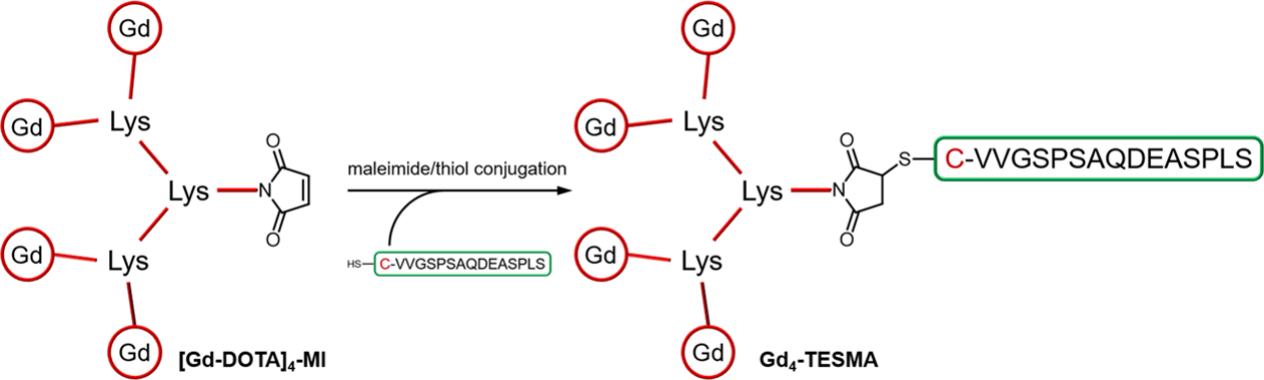
Synthetic approach to Gd_4_-TESMA.

Here, we report a new and improved version of the tropoelastin-specific MRI probe aimed at improving the pharmacokinetics, increasing the sensitivity for MRI detection, and extending the duration of the imaging window. To achieve that, we linked four Gd-chelates to the same tropoelastin recognizing peptide moiety to accumulate multiple copies of the gadolinium moiety within a single binding event. A lysine-based tetramer containing four Gd-DOTA monoamide chelates was conjugated to the tropoelastin-binding VVGS-peptide to obtain the compound Gd_4_-TESMA by adapting a synthetic approach previously described (Figure 1).^28,29^

The tetrameric imaging reporter was designed as a flexible molecule for minimizing binding interference to the biological target. Moreover, increasing the molecular size of the imaging probe could improve pharmacokinetics in plaques, potentially allowing for a longer imaging time window. Interestingly, the increased density of gadolinium at the biological target may pave the way for imaging atherosclerosis using techniques other than MRI, such as the novel spectral photon counting computed tomography (SPCCT). This is an X-ray technique that can be tailored to the selective detection of gadolinium by its K-edge specific absorption.^30−32^

## Results

### Synthesis and Characterization of Gd_4_-TESMA

The general synthetic approach to Gd_4_-TESMA relied on the synthesis of a tetrameric Gd-DOTA-like chelate functionalized with a maleimide function (compound [Gd-DOTA]_4_-MI). This gadolinium tetramer was then conjugated to a cysteine residue added at the N-terminus of the tropoelastin specific binding sequence (C-VVGSPSAQDEASPLS) through the thiol/maleimide reaction to yield the final Gd_4_-TESMA imaging probe (Figure 1).

**Figure 2:**
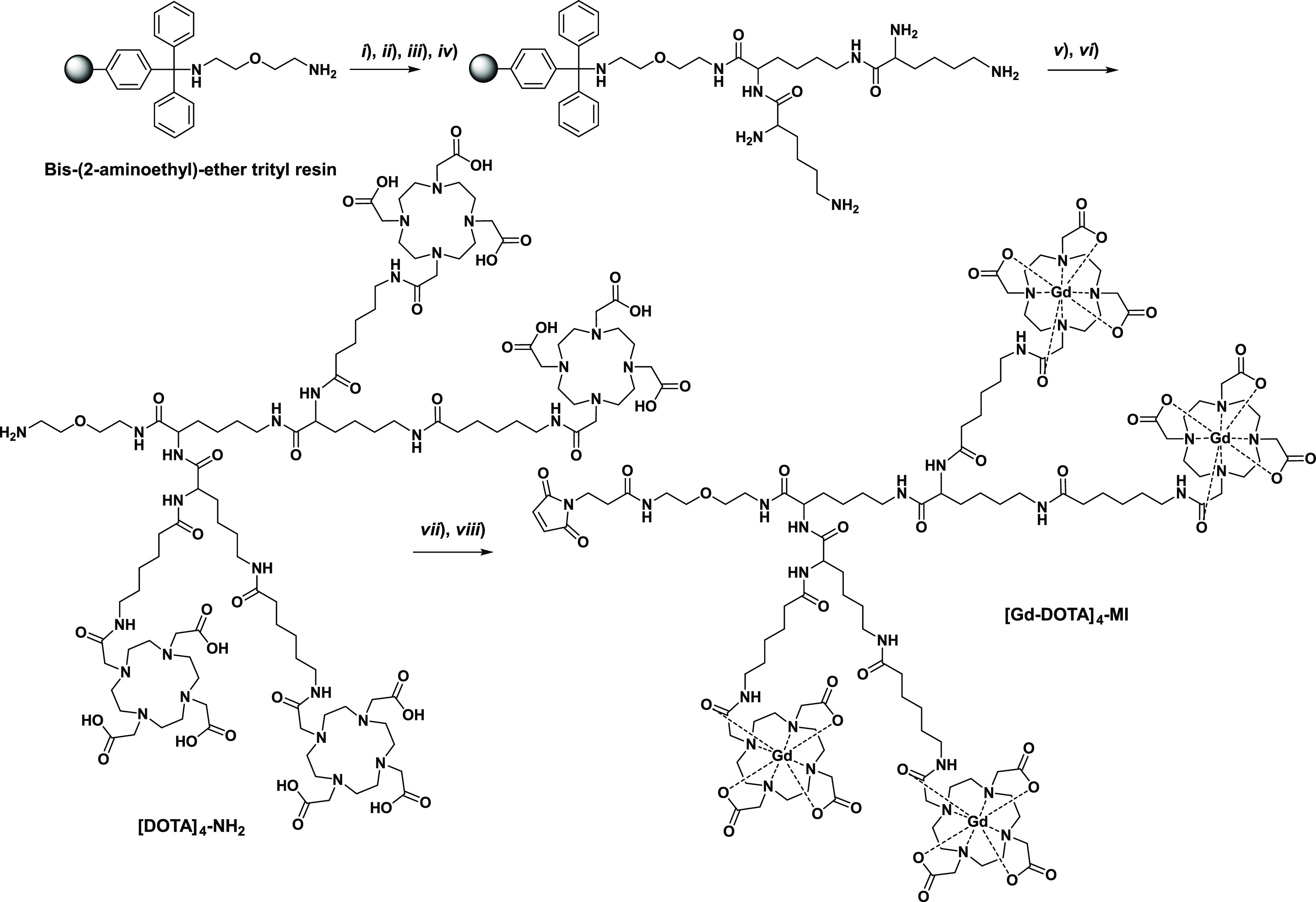
Synthesis of [Gd-DOTA]_4_-MI. (*i*) Fmoc-Lys(Fmoc)-OH, PyBOP, DIPEA, DMF, 1 h, 20% acetic anhydride; (*ii*) 40% piperidine-DMF (5 min) and 20% piperidine-DMF (15 min); (*iii*) Fmoc-Lys(Fmoc)-OH, PyBOP, DIPEA, DMF,1 h; (*iv*) 40% piperidine-DMF (5 min) and 20% piperidine-DMF (15 min); (*v*) DOTA(*t*Bu)_3_CONH(CH_2_)_5_COOH, PyBOP, DIPEA, DMF, 2 h ; (*vi*) TFA, DCM, TIS 49:49:2 (20 min) and TFA 100% (12 h); (*vii*) GdCl_3_ in water; and (*viii*) *N*-succinimidyl-3-maleimidopropionate in 50 mM phosphate buffer.

The tetrameric chelator based on DOTA (compound [DOTA]_4_-NH_2_) was synthesized by a solid phase peptide synthesis (SPPS) approach. This approach relies on (*i*) the attachment of the first lysine to a solid polymer by a covalent bond, (*ii*) the addition of two more lysines followed by the four DOTA-based chelating cages, and (*iii*) the final removal of the compound from the solid support (Figure 2).

The bis(2-aminoethyl)ether trityl resin and 9-fluorenylmethyloxycarbonyl (Fmoc) protection chemistry were chosen for the setup of the synthesis. After coupling the first lysine residue (under the form of Nα,Nε-di-Fmoc-l-lysine) to the resin, the resin was treated with acetic anhydride in DMF to acetylate the residual free amino groups exposed on its surface. After the removal of Fmoc by the addition of piperidine in DMF, two more lysine residues were attached. The coupling reactions were performed with 2.5-fold excess Fmoc-Lys(Fmoc)-OH in DMF, with PyBOP as the activator and DIPEA as the base. The branched tri-lysine core anchored on the resin provided four amino groups (two α-amino and two side-chain ε-amino groups) for attachment of the DOTA-like chelators. The latter coupling was performed with a small excess of the DOTA derivative DOTA(*t*Bu)_3_CONH(CH_2_)_5_COOH, whose synthesis has been described previously.^33^ The coupling reaction between DOTA and the tri-lysine scaffold turned out to be a tricky step, as the final yield was strongly dependent upon the activating agent. PyBOP as the activator was clearly superior to HBTU. The latter activator showed decreased yields because of the transfer of a tetramethyluronium moiety from HBTU to a lysine amino group, with the formation of a stable tetramethylguanidine derivative.^29,34^ Cleavage from the resin and purification by semipreparative HPLC afforded [DOTA]_4_-NH_2_ with an overall yield of about 60% and purity of 98% (λ = 220 nm). The Gd(III)-complex (compound [Gd-DOTA]_4_-NH_2_) was prepared by mixing stoichiometric amounts of [DOTA]_4_-NH_2_ and GdCl_3_ solution (1:4 ratio). After complexation, the amine end-group of the molecular structure of [Gd-DOTA]_4_-NH_2_ was easily converted into maleimide (compound [Gd-DOTA]_4_-MI) by reacting the tetrameric compound with a 3-fold molar excess of *N*-succinimidyl-3-maleimido propionate in phosphate buffer 50 mM and acetonitrile (3:1 ratio). Since some loss of the functionalized compound may take place because of the hydrolysis of the maleimide group in the aqueous solution, we optimized the reaction to condition such to have total conversion within 1 h by carefully maintaining pH at 6.5. UPLC-UV-MS (ESI+) analysis after the removal of excess reagents and salts by size exclusion chromatography showed a purity >88% (λ = 220 nm).

The tropoelastin binding sequence, ending up with a cysteine residue at the N-terminus, was synthesized by standard SPPS by means of the Fmoc chemistry with 2-chlorotrityl chloride resin. The fully deprotected peptide was purified by semipreparative HPLC (98% purity) and then was reacted with [Gd-DOTA]_4_-MI at a molar ratio of 1:1 at pH 6.5 to yield the final product Gd_4_-TESMA. After a final purification step by semipreparative HPLC, an overall yield of about 60% and purity >95% (λ = 220 nm) were achieved. It is worth emphasizing that, by such a procedure, a purified, preformed tetrameric Gd(III)-chelate is directly conjugated to the targeting vector. This approach eliminates the potential issue of ″free″ gadolinium, *i.e.,* the presence of Gd(III) ions being weakly coordinated to secondary coordinating sites potentially available in the peptide structure.

### Relaxivity Studies

**Figure 3:**
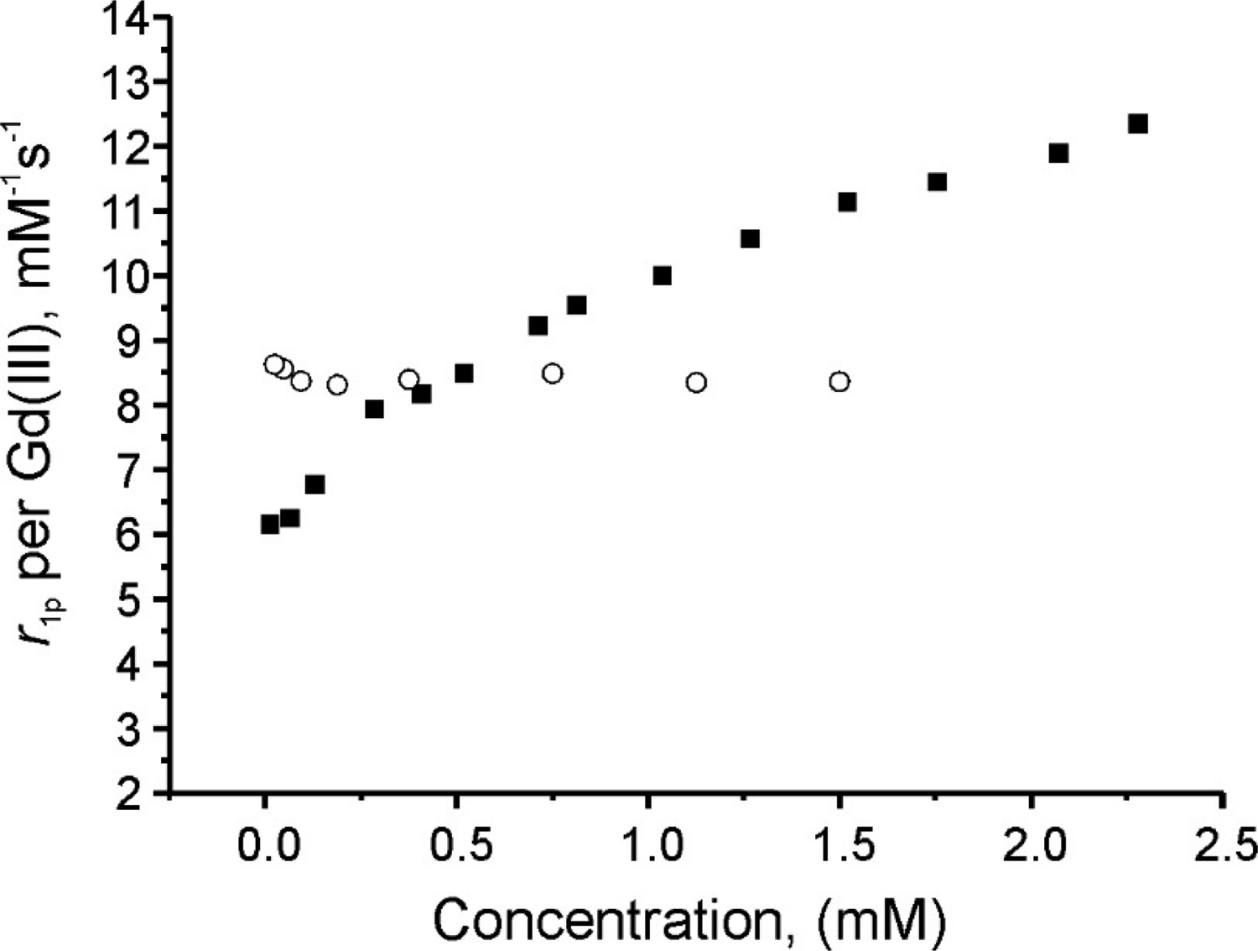
Plot of the millimolar relaxivity per gadolinium *r*_1p_ of Gd-TESMA (solid squares) and Gd_4_-TESMA (open circles) as a function of the probe concentration (20 MHz, 298 K, 3.8 mM HEPES, 150 mM NaCl, pH 7.2).

The relaxivity per gadolinium of Gd_4_-TESMA is 8.5 ± 0.2 mM^–1^ s^–1^ (at 20 MHz, 298 K, 3.8 mM HEPES, 150 mM NaCl, pH 7.2; Table 1), while that of Gd-TESMA under the same conditions cannot be defined uniquely as it changes with the concentration of the complex. Namely, it increases from 6.1 mM^–1^ s^–1^ at 18 μM to 12.4 mM^–1^ s^–1^ at 2.3 mM (Figure 3).

**Table 1:** Relaxivities of Gd_4_-TESMA and Gd-TESMA at 20 and 125 MHz (298 K, 3.8 mM HEPES, 150 mM NaCl, pH 7.2)

compound	*r*_1_^mM^ (mM^–1^ s^–1^) at 20 MHz	*r*_1_^mM^ (mM^–1^ s^–1^) at 125 MHz
Gd_4_-TESMA		
per molecule	34.0 ± 0.8	32.4 ± 0.8
per Gd(III)	8.5 ± 0.2	8.1 ± 0.2
Gd-TESMA (1 mM)	10.0 ± 0.3	10.4 ± 0.3

**Figure 4:**
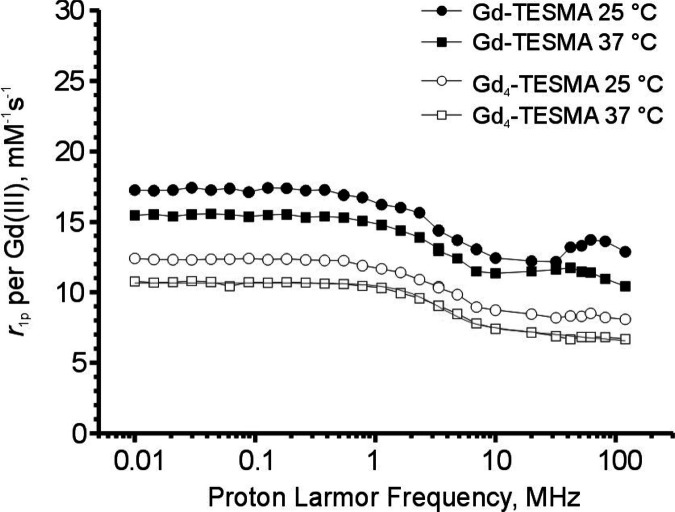
^1^H NMRD profiles showing the millimolar relaxivity per gadolinium *r*_1p_ of Gd-TESMA (2.3 mM, solid symbols) and Gd_4_-TESMA (1.3 mM, open symbols) in the HEPES buffer (pH 7.2) at 298 K (circles) and 310 K (squares). The solid line represents the best fitting of the experimental points at 310 K to the SBM and Freed theory of paramagnetic relaxation. The following parameters were kept fixed: water diffusion coefficient (*D*) = 3.1 10^–5^ cm^2^ s^–1^; distance between Gd(III) and the inner sphere water protons (*r*) = 3.1 Å; number of inner sphere water molecules (*q*) = 1; and distance of the closest approach of outer-sphere water protons = 3.8 Å. Curve fitting converged to the following: correlation time for molecular reorientation (τ_R_) = 170 ps; residence lifetime of the inner sphere water molecule (τ_M_) = 460 ns; correlation time for electronic relaxation (τ_v_) = 47 ps; and zero-field splitting energy (Δ^2^) = 1.3 10^19^ s^–2^.

Such behavior can be explained by the formation of polydispersed aggregates, whose average size and size distribution progressively increase with the concentration. It is worth noting that a similar dependence of the relaxivity with the concentration was observed for other peptidic probes having a closely related structure.^35^ The ^1^H nuclear magnetic resonance dispersion (NMRD) profiles of Gd_4_-TESMA and Gd-TESMA were acquired to get more insights into the factors driving their relaxivity (Figure 4).

Despite its molar mass of about 4.8 kDa, the ^1^H NMRD profile of Gd_4_-TESMA shows a monotonic single dispersion that is typical of low molar mass Gd-DOTA monoamide compounds characterized by fast molecular tumbling motions.^36^ Multiparametric fitting of the ^1^H NMRD profile at 310 K yielded an estimate of the molecular correlation time for molecular tumbling (τ_R_) of 170 ps according to the Solomon–Bloembergen–Morgan (SBM) theory for inner sphere paramagnetic relaxation combined with the Freed theory for the outer sphere contribution.^37−39^ This value is between that of a true low molar mass Gd-DOTA monoamide complex (about 80 ps)^36^ and that expected for a rigid system with 5 kDa molar mass (of the order of nanoseconds)^37^ and comparable to that of other multimeric Gd-DOTA monoamide based compounds (240 ps).^40^ This can be ascribed to the high flexibility of the linkers connecting the paramagnetic centers to the peptide moiety, which decouples local motions of the Gd(III) chelating cages from global molecular reorientation motions.^28,35,41^ The ^1^H NMRD profile of Gd-TESMA (at 2.3 mM concentration) showed a weak but still appreciable relaxivity peak centered at about 40 MHz corresponding to slow tumbling systems (*i.e.,* systems having a large size). Although such a relaxivity peak was much smaller compared with truly rigid macromolecular systems (*e.g.,* nanosystems),^42^ it confirmed that the system self-assembled into aggregates with high polydispersity at relatively high concentrations. The ^1^H NMRD profile can be thought as arising from the contribution of species spanning from monomers to aggregates having a heterogeneous dispersion of molecular size that cannot be accounted for by our fitting model. As a matter of fact, the standard SBM model did not converge to a meaningful fit of the data.

### *In Vitro* Binding Affinity

**Figure 5:**
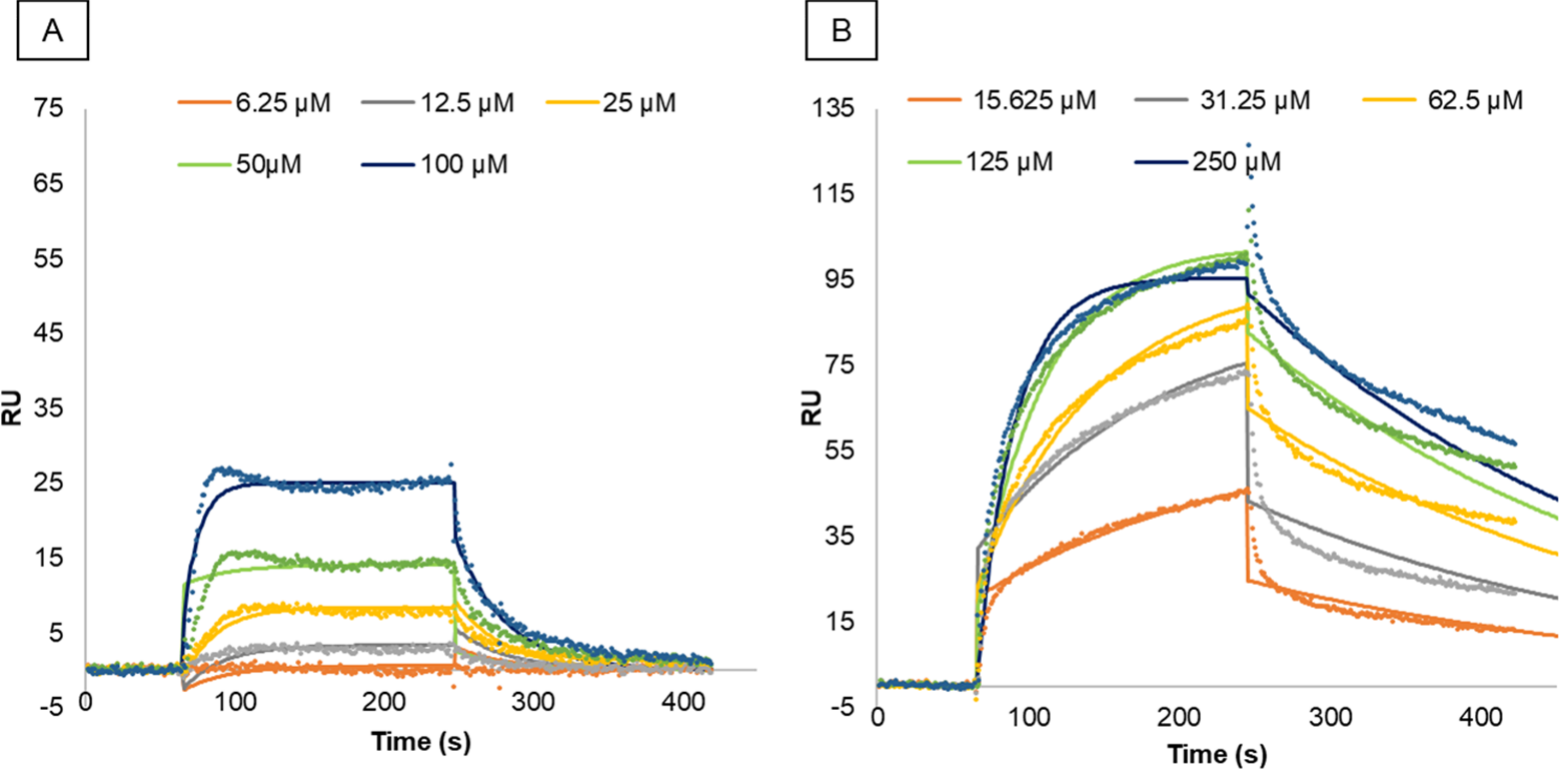
Representative SPR sensorgrams of the interaction between tropoelastin and Gd-TESMA (A) and Gd_4_-TESMA (B). The experimental data are shown as dots after subtraction of the signal from the control flow cell. Solid lines represent best-fit curves according to a 1:1 Langmuir binding model.

The binding affinity of Gd-TESMA and Gd_4_-TESMA to tropoelastin was assessed by surface plasmon resonance (SPR) under the kinetic affinity mode. The sensorgrams of Gd-TESMA and Gd_4_-TESMA at their increasing concentrations indicated a good interaction of both ligands with the immobilized tropoelastin (Figure 5). The fitting of sensorgram data to the 1:1 binding model allowed the estimation of the equilibrium dissociation constant *K*_D_ for Gd-TESMA (28 ± 12 μM) and Gd_4_-TESMA (41 ± 12 μM), confirming similar binding affinities of the two probes to tropoelastin (Table 2). Higher association (*k*_a_) and dissociation (*k*_d_) rate constants were estimated for Gd-TESMA as compared to Gd_4_-TESMA; this was likely related to the difference of their molar masses.

**Table 2:** Association Rate Constant *k*_a_, Dissociation Rate Constant *k*_d_, and Equilibrium Dissociation Constant *K*_D_ Characterizing the Interaction between Immobilized Human Recombinant Tropoelastin and Gd-TESMA/Gd_4_TESMA^a^

analyte	*k*_a_ (M^–1^ s^–1^)	*k*_d_ (s^–1^)	*K*_D_ (μM)
Gd-TESMA	880 ± 160	2.3 × 10^–2^ ± 0.5 × 10^–2^	28 ± 12
Gd_4_-TESMA	94 ± 40	3.4 × 10^–3^ ± 0.3 × 10^–3^	41 ± 12

a Values are mean ± SD from triplicate measurements.

### Serum Stability Assays

**Figure 6:**
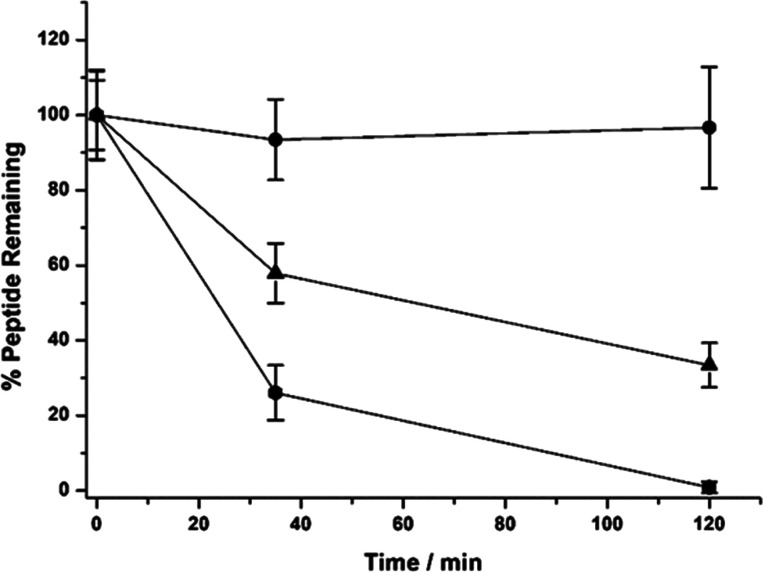
Serum stability profiles of Gd_4_-TESMA (circles), the VVGS-peptide (triangle), and a positive control peptide (square).

The stabilities of Gd_4_-TESMA and the parent VVGS-peptide in human serum were evaluated by HPLC (Figure 6). Compounds were incubated up to 120 min, and aliquots were taken at different times: 0, 35, and 120 min. The VVGS-peptide showed a half-life in serum of about 45 min. After 2 h, about 20% of the VVGS-peptide remained in the serum. On the other hand, Gd_4_-TESMA could be recovered unchanged after 2 h of incubation. This demonstrates a very good stability in serum both toward the proteolysis of the peptide moiety and toward the deconjugation of the thioether bond and binding to other serum thiols.^43^ It is likely that the conjugation with the tetrameric gadolinium chelate prevented peptide digestion mediated by the N-exopeptidases in the blood.

### *In Vivo* MRI of Atherosclerotic Plaques in the ApoE^–/–^ Mouse Model Using Gd_4_-TESMA at a Clinical Field

**Figure 7:**
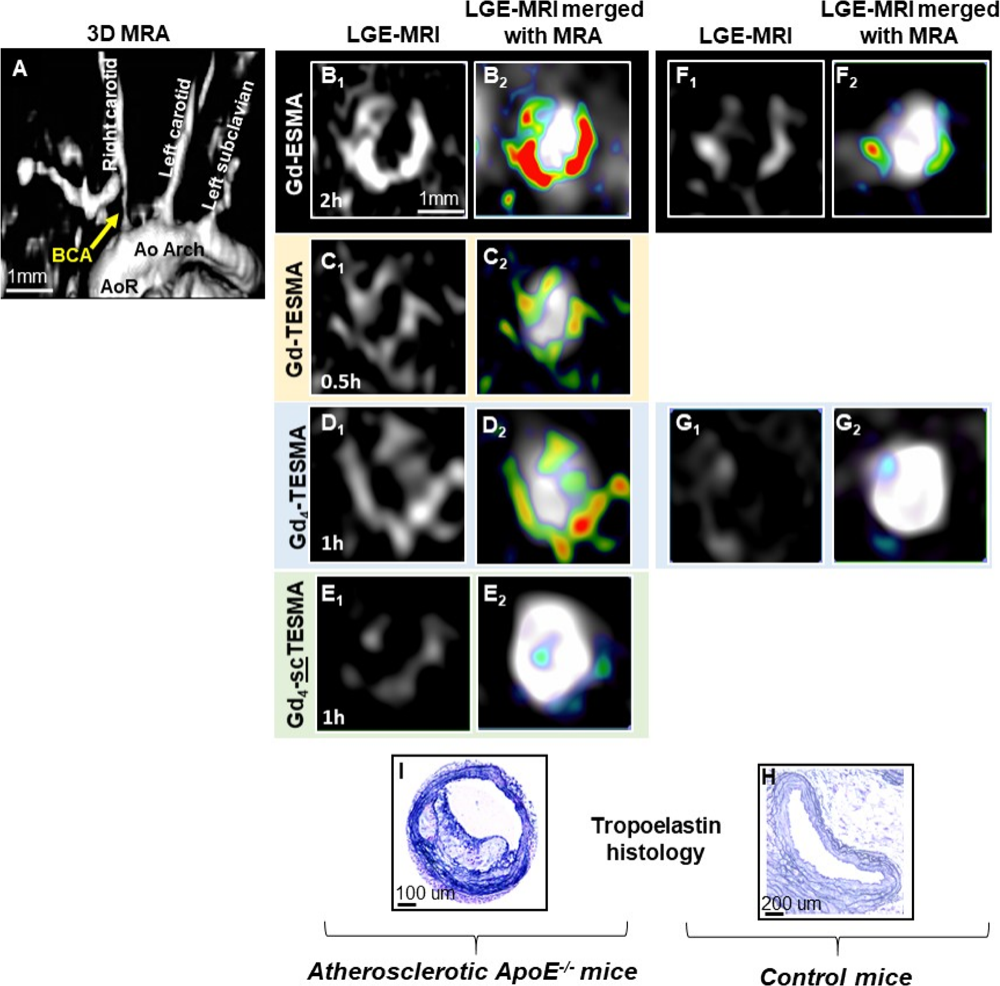
Molecular imaging of tropoelastin in atherosclerotic ApoE^–/–^ and control mice using the tetrameric Gd_4_-TESMA contrast agent. (A) The 3D reconstructed angiogram (MRA) shows the vasculature. Late gadolinium enhancement (LGE) images (B_1_–D_1_) and merged LGE and angiography images (B_2_–D_2_) compare the signal enhancement of atherosclerotic plaques located in the brachiocephalic artery of the same animal imaged serially after the administration of the elastin-binding Gd-ESMA (2 h), the tropoelastin-binding Gd-TESMA (0.5 h), and the tropoelastin-binding tetrameric Gd_4_-TESMA (1 h) agents. (E_1_, E_2_) LGE and merged images show a low uptake of the tetrameric tropoelastin-binding agent attached to a scrambled peptide (Gd_4_-scTESMA). (F_1_, F_2_) LGE and merged LGE and angiography images show signal enhancement of the brachiocephalic artery of control mice after the injection of the elastin agent Gd-ESMA but (G_1_, G_2_) no enhancement after the injection of the tropoelastin agent Gd_4_-TESMA. Immunohistochemistry using a tropoelastin antibody shows the absence of tropoelastin in control tissues (H) and dense tropoelastin molecules (dark/purple signal) in the disease artery (I).

The Gd_4_-TESMA probe was assessed for its ability to enhance the MRI signal within plaques in the brachiocephalic artery of an ApoE^–/–^ murine model of plaque progression and in control nonatherosclerotic mice. For these studies, atherosclerotic ApoE^–/–^ mice were scanned after the tandem administration of Gd-ESMA, Gd-TESMA (24 h after the Gd-ESMA administration), and finally Gd_4_-TESMA (24 h after the Gd-TESMA administration; Supporting Information Figure S1). Previous studies have shown that 24 h is sufficient to ensure the complete washout of Gd-ESMA and Gd-TESMA from the blood, plaques, and vessel walls.^9^ All molecular probes were injected at a dose of 0.2 mmol kg^–1^ (corresponding to the same dose of injected gadolinium for Gd-ESMA and Gd-TESMA and to a dose of 0.8 mmol_Gd_ kg^–1^ for Gd_4_-TESMA). A 3D reconstructed MR angiogram shows the vasculature extending from the aortic root to the carotid arteries that defines the imaging volume (Figure 7A). Late gadolinium enhanced (LGE) inversion recovery *T*_1_-weighted images acquired at every 30 min postinjection of each agent allowed for the accurate detection and localization of atherosclerotic plaques and elastin/tropoelastin remodeling in the brachiocephalic artery. LGE images after administration of the Gd-ESMA agent in ApoE^–/–^ mice showed a strong circumferential enhancement of the plaque surrounding the lumen (Figure 7B1,2) that persisted up to 2 h postinjection. LGE images after the administration of the monomeric Gd-TESMA probe showed an enhancement of the same segment of the vessel wall. However, the area of the enhancement was less compared to that observed with Gd-ESMA, and the signal peaked at 0.5 h postinjection (Figure 7C1,2). Compared to Gd-TESMA, Gd_4_-TESMA showed a stronger enhancement of the plaque that peaked at 1 h postinjection (Figure 7D1,2). To check whether the uptake and retention within the plaques were specific of the targeting sequence, control experiments were carried out by using a probe containing a scrambled peptide sequence that is not able to recognize tropoelastin (Gd_4_-scTESMA probe). The scrambled Gd_4_-scTESMA showed low/negligible accumulation within plaques in comparison to Gd_4_-TESMA (Figure 7E1,2).

LGE images of the brachiocephalic artery acquired from healthy animals showed that Gd-ESMA delineates the vessel wall because it binds to cross-linked elastin that is endogenously present in healthy vessel wall (Figure 7F1,2). Importantly, injection of Gd_4_-TESMA in heathy mice showed little accumulation of the agent within healthy vessel walls that are known to contain polymeric elastin but not monomeric tropoelastin (Figure 7G1,2). This conclusion drawn from the observed MRI signal was validated by immunohistochemistry of the excised tissues using a tropoelastin-specific antibody. There was a lack of immune-positive tropoelastin fibers in the control vessel wall (Figure 7H), whereas a dense tropoelastin network (black/purple staining) was observed within the atherosclerotic plaque (Figure 7I).

**Figure 8:**
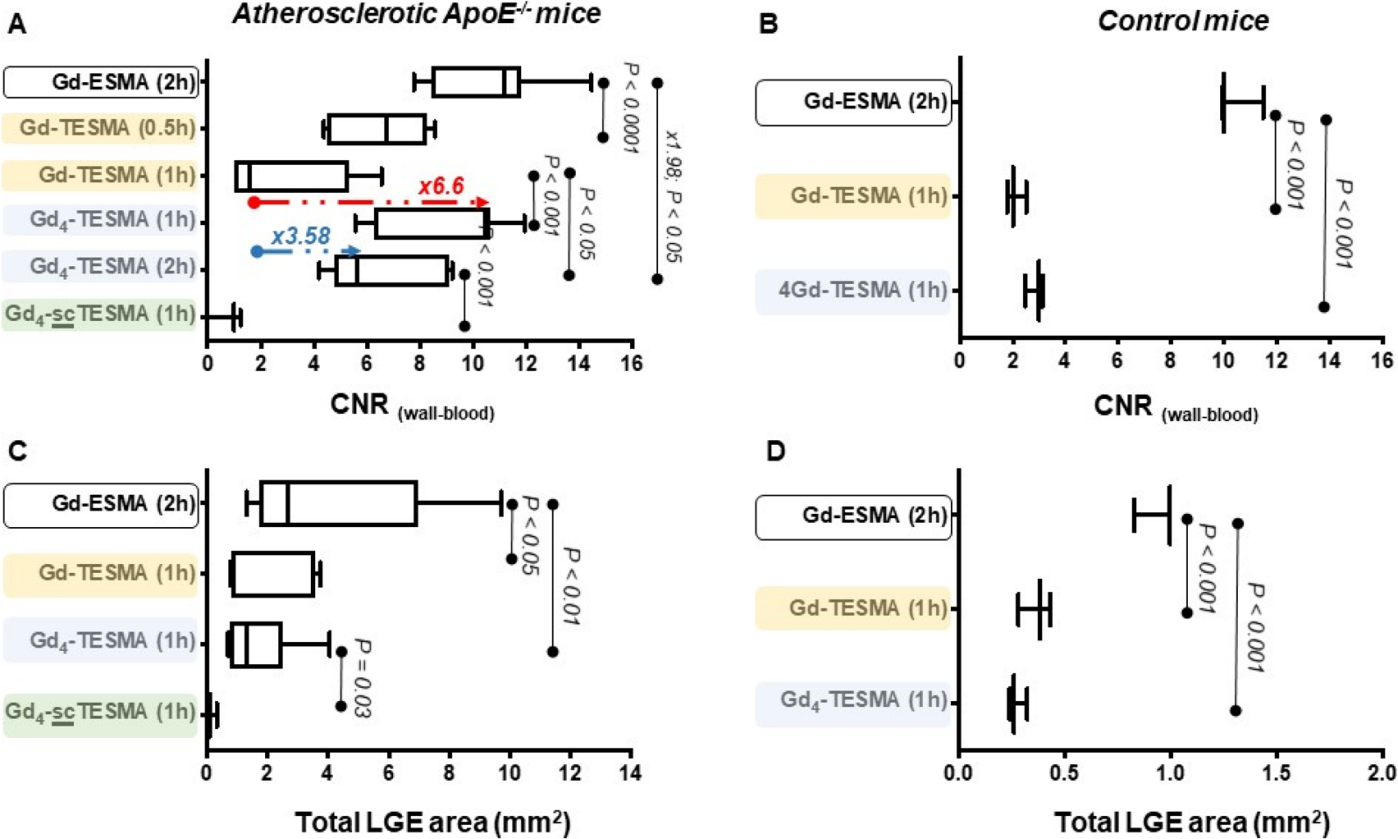
Quantitative assessments of the MRI measurements. Vessel wall-to-blood CNR at several time points after the injection of Gd-ESMA, Gd-TESMA, Gd_4_-TESMA, and Gd_4_-scTESMA (scrambled sequence as a negative control) in atherosclerotic ApoE^–/–^ mice (A) and control mice (B). Total area showing late gadolinium enhancement (LGE) in atherosclerotic ApoE^–/–^ mice (C) or control mice (D).

Quantitative assessments of the MRI measurements in ApoE^–/–^ and control mice are shown in Figure 8. All three compounds showed specific uptake in atherosclerotic plaques of ApoE^–/–^ mice (Figure 8A) but with clear differences in wall-to-blood CNR. Gd-ESMA had a peak CNR of 11.17 (interquartile range, IQR = 8.4–11.8) 2 h postinjection; Gd-TESMA had a maximum CNR of 6.75 (IQR = 4.5–8.2) at 0.5 h; and Gd_4_-TESMA had a maximum CNR of 10.47 (IQR = 6.3–10.6), 1.57-fold higher than that of Gd-TESMA, at 1 h. While the CNR of Gd-TESMA was significantly decreased 1 h postinjection (CNR = 1.58, IQR = 1.0–5.3), that of Gd_4_-TESMA was more persistent up to 2 h (CNR = 5.66, IQR = 4.8–9.0), overall extending the duration of the imaging window from 0.5 to 2 h. The vessel wall CNR measurements in control animals (Figure 8B) were significantly higher 2 h after the administration of Gd-ESMA compared to 1 h after the injection of Gd-TESMA and Gd_4_-TESMA, demonstrating that only Gd-ESMA can bind to the cross-linked elastin found in the control vessel (CNR = 10, IQR = 9.9–11.5). Simultaneously, it demonstrates the specificity of the Gd-TESMA (CNR = 2.0, IQR = 1.8–2.5) and Gd_4_-TESMA (CNR = 3.0, IQR = 2.48–3.1) probes to only bind to non-cross-linked tropoelastin that is absent in control vessels.

In ApoE^–/–^ mice, Gd-ESMA showed the largest enhancement area with 2.69 mm^2^ (IQR = 1.7–6.9), while Gd-TESMA and Gd_4_-TESMA had smaller but comparable values of 0.87 mm^2^ (IQR = 0.78–3.56) and 1.3 mm^2^ (IQR = 0.77–2.48), respectively (Figure 8C). The scrambled Gd_4_-scTESMA showed low/negligible accumulation within plaques in comparison to Gd_4_-TESMA in terms of enhanced area (0.1 mm^2^, IQR = 0–0.31). Similar to the CNR, the area of enhancement after administration of Gd-ESMA with 0.99 mm^2^ (IQR = 0.82–0.99) was the largest compared with that measured after the injection of Gd-TESMA (0.38 mm^2^, IQR = 0.28–0.43) and Gd_4_-TESMA (0.26 mm^2^, IQR = 0.24–0.32) in control animals (Figure 8D). This is consistent with the fact that atherosclerotic plaques contain a mixture of mature elastin and tropoelastin, and Gd-ESMA is known to bind to both biological targets.^20^ Conversely, Gd-TESMA and Gd_4_-TESMA bind only to the subset of tropoelastin molecules within the plaque.

## Discussion

Gd-TESMA has been successfully employed for tropoelastin specific MR imaging by virtue of its ability to discriminate between tropoelastin and mature, cross-linked elastin. Tropoelastin imaging enables the *in vivo* assessment of dysfunctional elastogenesis that is in turn an emerging biomarker of atherosclerotic diseases. Tropoelastin provided a marker for MRI of atherosclerotic plaque progression and instability.^9^ It was also correlated with the development and rate of aortic expansion of aortic aneurysm.^27^

The scope of this study was to enhance the imaging properties of the Gd-TESMA probe to enable improved MR tropoelastin imaging (*e.g.,* higher CNR and prolonged imaging window). The chosen strategy to achieve that goal was to link four paramagnetic gadolinium chelates to the tropoelastin specific VVGS-peptide to increase the relaxivity per molecule roughly by a factor of 4. The chelate chosen to build up the tetrameric reporter unit is of the Gd-DOTA-monoamide type, as this macrocyclic ligand is known to have high thermodynamic, kinetic, and metabolic stability that minimizes transmetallation and gadolinium release issues. It is therefore preferred to other types of chelates for clinical translation.^44,45^

The relaxivity per molecule of Gd_4_-TESMA (HEPES saline buffer, pH 7.2, 298 K, 20 MHz) is 3.4-fold greater than that of Gd-TESMA (at 1.0 mM gadolinium concentration). This ratio is slightly smaller than the expected factor of 4 because of two reasons: (*i*) the high flexibility of the Gd_4_-TESMA tetrameric moiety, avoiding the macromolecule effect on the relaxivity of Gd_4_-TESMA, and (*ii*) the aggregation of Gd-TESMA, but not of Gd_4_-TESMA, into molecular assemblies that in turn results into a slight concentration-dependent macromolecule effect.

The relaxivity per gadolinium (8.5 ± 0.2 mM^–1^ s^–1^) is in line with that of the free form of other multimeric Gd-DOTA-monoamide complexes based on a flexible scaffold.^28,40^ While Gd(III)-DOTA-monoamide multimers designed with rigid or cross-linked structures in the bound form are known to have a moderate relaxivity enhancement in comparison to the free form (macromolecule effect), Gd_4_-TESMA is expected to have a limited relaxivity enhancement in the tropoelastin bound form because of the high flexibility of the tetramer moiety. While sacrificing the small relaxivity increase due to the macromolecule effect, flexibility was introduced in the probe design to minimize potential steric hindrance effects due to the bulky tetrameric Gd-DOTA moiety of the probe. Indeed, a good binding affinity between Gd_4_-TESMA and the biological target, tropoelastin, was maintained. Kinetic affinity measurements by SPR showed no substantial differences between the dissociation constants of Gd-TESMA and Gd_4_-TESMA.

In the atherosclerotic ApoE^–/–^ mouse model, both Gd-TESMA and Gd_4_-TESMA were specifically retained in diseased atherosclerotic animals having plaques rich in tropoelastin. The specificity for such tropoelastin-rich regions was supported by two different control experiments: the scrambled version of Gd_4_-TESMA showing negligible accumulation within plaques and the Gd_4_-TESMA not accumulating in the healthy vessel wall, as previously also demonstrated for the monomeric Gd-TESMA.^9^ However, Gd_4_-TESMA and Gd-TESMA showed a clear difference in their times required to reach the maximum CNR (Figure 8A). Gd-TESMA reached the maximum CNR in half an hour and then significantly decreased within 1 h. Conversely, Gd_4_-TESMA reached the maximum CNR in 1 h p.i. and the CNR decreased by 46% after 2 h. Importantly, the CNR of Gd_4_-TESMA at 2 h p.i. was still as high as the maximum CNR of Gd-TESMA at 0.5 h. This difference in the pharmacokinetics reveals a clear benefit of Gd_4_-TESMA with respect to Gd-TESMA, allowing for a better delineation between the vessel wall and blood, increasing the sensitivity, and widening the imaging time window.

The Gd_4_-TESMA was rationally designed to image elastin related fibrosis with an eye to clinical translation. The DOTA chelator used in Gd_4_-TESMA is currently used in the most stable commercially available MRI contrast agent Gd-DOTA for which there are no reported cases of nephrogenic systemic fibrosis. The higher sensitivity and vessel wall delineation achieved by Gd_4_-TESMA may potentially allow for lowering the injected dose, thereby reducing both manufacturing costs and potential gadolinium effects. These improvements in vessel wall imaging made by using Gd_4_-TESMA combined with imaging protocols, such as the inversion recovery for late gadolinium enhancement, that are available on clinical scanners and have been used for imaging the heart and thrombosis in patients may facilitate the future use of the molecular imaging probe Gd_4_-TESMA for imaging dysfunctional elastogenesis in patients.

## Conclusions

The modular and scalable chemistry of Gd_4_-TESMA, its immediate target localization after injection, its fast background washout, the increased signal enhancement, the elongated image acquisition window, and its high stability toward proteolysis and deconjugation in serum make Gd_4_-TESMA a desirable probe for molecular imaging of dysfunctional/dysregulated elastogenesis. The high CNR achieved by Gd_4_-TESMA increases the sensitivity for the detection of disease and treatment response, overcoming this major limitation of molecular MRI. It allows taking advantage of the higher spatial resolution, non-ionizing radiation, excellent soft tissue contrast, multiple image contrasts, and advanced motion correction protocols of MRI.

## Experimental Section

### General Methods

Bis(2-aminoethyl)ether trityl resin, PyBOP (benzotriazol-1-yloxytripyrrolidinophosphonium hexafluorophosphate), Fmoc-Lys(Fmoc)-OH, and all other Fmoc-AA-OH were purchased from Novabiochem (Darmstadt, Germany). Gd-TESMA was purchased from For Peptide Protein Research Limited (Fareham, UK). All other chemicals were purchased from Sigma-Aldrich. NMR spectra were recorded at 310 K on a Bruker AVANCE 600 spectrometer operating at 14 T (corresponding to 600 and 150 MHz ^1^H and ^13^C Larmor frequencies, respectively). Analytical and preparative HPLC–MS was carried out on a Waters AutoPurification system (3100 Mass Detector, 2545 Pump Gradient Module, 2767 Sample Manager, and 2998 PDA detector). ESI+ mass spectra were calculated as the exact mass by considering the ^158^Gd isotope for gadolinium complexes. UPLC analysis was performed using a UPLC Acquity H-Class coupled with the QDa and TUV detectors. All compounds are >95% pure by HPLC.

### Synthesis of [DOTA]_4_-NH_2_

The synthesis of [DOTA]_4_-NH_2_ was performed on the solid phase manually by using bis(2-aminoethyl)ether trityl resin (0.96 mmol; 1.95 g) in a peptide synthesis vessel. After swelling in dimethylformamide (DMF) (25 mL) for 10 min, Fmoc-Lys(Fmoc)-OH (2.39 mmol; 1.41 g), PyBOP (2.39 mmol; 1.24 g), and *N*,*N*-diisopropylethylamine (DIPEA)(4.78 mmol; 0.83 mL) in DMF (20 mL) were added and the reaction slurry was shaken at room temperature for 1 h followed by a capping reaction with 20% acetic anhydride in DMF (20 mL). The resin was washed with DMF (3 × 25 mL) and then treated with 20 mL 40% piperidine/DMF (5 min) and 20 mL 20% piperidine/DMF solution for 15 min (× 2) to remove Fmoc protecting groups. After washing with DMF, the coupling cycle was repeated using a double quantity of Fmoc-Lys(Fmoc)-OH (4.78 mmol; 2.82 g), PyBOP (4.78 mmol; 2.49 g), and DIPEA (9.55 mmol; 1.67 mL) in DMF (20 mL) followed by piperidine treatment. After washing with DMF, a mixture of DOTA(*t*Bu)_3_CONH(CH_2_)_5_COOH^33^ (4.02 mmol; 2.76 g), PyBOP (4.01 mmol; 2.09 g), and DIPEA (8.02 mmol; 1.40 mL) in DMF (20 mL) was added and the reaction was shaken at room temperature for 2 h. The resin was washed with DMF (3 × 20 mL) and DCM (3 × 20 mL) and then treated with 10 mL of 50% TFA in DCM plus 2% of triisopropylsilane (TIS) for 20 min at room temperature. The TFA solution was collected, and the resin was washed with 10% TFA in DCM (5 mL). The combined extracts were concentrated under reduced pressure; then 20 mL of fresh TFA was added, and the mixture was stirred for 12 h to ensure the complete removal of *t*Bu from DOTA chelators. The cleavage solution was concentrated under reduced pressure, and the crude product was precipitated in cold Et_2_O (20 mL) and purified by semipreparative RP-HPLC by using a Waters XTerra prep RPdC8 column, 5 μm, 19 × 100 mm, with a gradient of CH_3_CN (0.1% TFA) in H_2_O (0.1% TFA) from 5 to 10% in 3 min and from 10 to 35% in 12 min (20 mL min^–1^). The pure product was obtained as a TFA salt (white powder) by lyophilization. The pure product was obtained as a white powder (1.2 g, 50% of yield). The purity of the tetrameric compound was verified by analytical UPLC using an ACQUITY UPLC Peptide BEH C18 column, 1.7 μm, 2.1 × 150 mm, applying a gradient of CH_3_CN (0.05% TFA) in H_2_O (0.05% TFA) from 5 to 20% in 10 min and from 20 to 80% in 12 min (0.4 mL min^–1^). The purity was >95% based on the chromatographic peak area revealed at 220 nm (retention time 3.7 min). ESI + MS *m/z* (calcd. for C_110_H_196_N_28_O_36_): [M + 3H]^3+^ 829.8 (obsd.), 829.8 (calcd.); [M + 4H]^4+^ 622.7 (obsd.), 622.6 (calcd.); [M + 5H]^5+^ 498.3 (obsd.), 498.3 (calcd.) See Supporting Information Figure S2 for UPLC-UV-ESI-MS and Figure S3 for ^1^H NMR.

### Synthesis of [Gd-DOTA]_4_-NH_2_

An equimolar amount of GdCl_3_·6H_2_O (0.24 mmol; 89.2 mg) was slowly added to the ligand solution (0.06 mmol; 149.2 mg) (5 mL). The pH of the solution was adjusted to 6.5 with NaOH, and the mixture was stirred overnight. The Gd-complex was then desalted by size exclusion chromatography on PD-10 Desalting Columns prepacked with Sephadex G25, using Milli-Q water as the mobile phase, and finally freeze-dried. The amount of residual free Gd^3+^ ions was assessed by the Orange Xylenol UV method.^46^ [Gd-DOTA]_4_-NH_2_ was found to contain less than 0.3% (mol/mol) of residual free Gd^3+^ ions. The purity of [Gd-DOTA]_4_-NH_2_ was checked by using the same analytical UPLC method described for [DOTA]_4_-NH_2_ (retention time 3.5 min). The purity was >95% (λ = 220 nm). ESI + MS *m/z* (calcd. for C_110_H_184_Gd_4_N_28_O_36_): [M + 3H]^3+^ 1035.6 (obsd.), 1035.3 (calcd.); [M + 4H]^4+^ 776.9 (obsd.), 776.9 (calcd.) (see Supporting Information Figure S4).

### Synthesis of [Gd-DOTA]_4_-MI

*N*-Succinimidyl-3-maleimido propionate (0.06 mmol; 16 mg) dissolved in 1 mL of CH_3_CN was slowly added to a solution of [Gd-DOTA]_4_-NH_2_ (0.02 mmol; 50 mg) in buffer phosphate (50 mM; 3 mL; pH 6.7). After stirring for 60 min at room temperature, the organic phase was evaporated and then desalted and purified by Sephadex G-25 in a PD-10 Desalting Column against water. The fractions containing the purified product were collected and lyophilized (36.6 mg, 73.2% of yield). The purity of [Gd-DOTA]_4_-NH_2_ was checked by using the same analytical UPLC method described for [DOTA]_4_-NH_2_ (retention time 4.5 min). The purity was >88% (λ = 220 nm). ESI + MS *m/z* (calcd. for C_117_H_189_Gd_4_N_29_O_39_): [M + 3H]^3+^ 1086.3 (obsd.), 1085.9 (calcd.); [M + 4H]^4+^ 814.5 (obsd.), 814.7 (calcd.) (Supporting Information Figure S5).

### Synthesis of the C-VVGS Peptide

The 16-mer peptide sequence CVVGSPSAQDEASPLS (C-VVGS peptide), containing the tropoelastin binding motif, was performed manually by standard Fmoc solid-phase peptide synthesis on the 2-chlorotrityl chloride resin (500 mg, 0.8 mmol, loading: 1.60 mmol/g). Each reaction step was performed at room temperature under gentle stirring. Amino acid coupling reactions were performed using 2.5 equiv of the Fmoc-protected amino acid, 2.5 equiv of the activating agent (PyBOP), and 5 equiv of the base (DIPEA) in DMF for 30 min. Fmoc removal was performed using a solution of 20% piperidine in DMF twice for 10 min. The resin was washed with DMF after each reaction step. Final cleavage and deprotection were performed with a solution of trifluoracetic acid (TFA)/H_2_O/phenol/triisopropylsilane (88:5:5:2, v/v/wt/v) for 3 h at room temperature. Et_2_O was added, and the precipitated crude product was collected and dried. The crude peptide was then dissolved in water and purified by semipreparative RP-HPLC by using a Waters XTerra prep RPdC8 column, 5 μm, 19 × 100 mm, with a gradient of CH_3_CN (0.1% TFA) in H_2_O (0.1% TFA) from 5 to 10% in 3 min and from 10 to 35% in 12 min (20 mL min^–1^) and lyophilized to give peptide as a white powder (0.62 g, TFA salt, 50% yield based on the estimated loading of the resin). The purity of the peptide was verified by analytical HPLC using an Atlantis C18, 3.5 μm, 4.6 mm × 150 mm column and 0.1% TFA in water (solvent A) and 0.1% TFA in acetonitrile (solvent B); elution was done with a linear 5 to 55% gradient of solvent B into A over 40 min at a 1 mL min^–1^ flow rate with UV detection at 220 and 254 nm (retention time = 14.8 min, purity >98%, λ = 220 nm). ESI + MS *m/z* (calcd. for C_63_H_102_N_17_O_27_S): [M + 2H]^2+^ 773.8 (obsd.), 774.3 (calcd.); [M + H]^2+^ 1547.3 (obsd.), 1547.6 (calcd.) (Supporting Information Figure S6). The scrambled peptide (C-GAESAPLVSSVQSPD) was synthetized following the same synthetic procedure of the C-VVGS peptide. HPLC analysis: retention time = 14.8 min, purity >98% (λ = 220 nm). ESI + MS *m/z* (calcd. for C_63_H_102_N_17_O_27_S): [M + 2H]^2+^ 773.9 (obsd.), 774.3 (calcd.); [M + H]^+^ 1547.5 (obsd.), 1547.6 (calcd.)

### Synthesis of Gd_4_-TESMA

A solution of the C-VVGS peptide (0.03 mmol; 47 mg) in acetate buffer (0.1 M; 2 mL; pH 6.7) was slowly added to a solution of [Gd-DOTA]_4_-MI (0.03 mmol; 100 mg) in water (2 mL) under an argon atmosphere. The mixture was stirred at room temperature for 1 h. The crude product was purified by preparative RP-HPLC using an Atlantis prep RPdC18 column, 5 μm, 19 × 100 mm, with a gradient of CH_3_CN in 10 mM ammonium acetate from 5 to 35% in 30 min (20 mL min^–1^). Finally, the purified product was desalted by gel permeation chromatography with Sephadex G10 and lyophilized (62 mg, 43% yield). The purity of Gd_4_-TESMA was evaluated by analytical HPLC using an Atlantis C18 column by the same method described for C-VVGS peptide. Retention time was 14.8 min, and the purity of the compound >95% (λ = 220 nm). ESI + MS *m/z* (calcd. for C_180_H_292_Gd_4_N_46_O_65_S): [M + 5H]^5+^ 962.1 (obsd.), 961.3; [M + 4H]^4+^ 1201.4 (obsd.), 1201.4 (calcd); [M + 3H]^3+^ 1602.0 (obsd.), 1601.6 (calcd) (Supporting Information Figure S7). The scrambled Gd_4_-scTESMA was synthetized following the same synthetic procedure of Gd_4_-TESMA. HPLC analysis: retention time = 15.0 min, purity >98% (λ = 220 nm). ESI + MS *m/z* (calcd. for C_180_H_292_Gd_4_N_46_O_65_S): [M + 5H]^5+^ 960.8 (obsd.), 961.3 (calcd.); [M + 4H]^4+^ 1201.4 (obsd.), 1201.4 (calcd); [M + 3H]^3+^ 1603.4 (obsd.), 1601.6 (calcd).

### Relaxometry

The water proton longitudinal relaxation rates as a function of the magnetic field strength were measured in aqueous solutions using a Fast Field-Cycling Stelar SmarTracer relaxometer (Stelar s.r.l., Mede (PV), Italy) over a continuum of magnetic field strengths from 0.00024 to 0.25 T (corresponding to 0.01–10 MHz proton Larmor frequencies). The relaxometer operates under computer control with an absolute uncertainty in 1/*T*_1_ of 1%. Additional longitudinal relaxation data in the range of 20–70 MHz were obtained using a Stelar Relaxometer connected to a Bruker WP80 NMR electromagnet adapted to variable-field measurements. The ^1^H *T*_1_ relaxation times were acquired by the standard inversion recovery method with a typical 90° pulse width of 3.5 μs, 16 experiments, and 4 scans. The temperature was controlled using a Stelar VTC-91 airflow heater equipped with a calibrated copper–constantan thermocouple (uncertainty of ±0.1 °C).

### Stability in Plasma

The serum stabilities of Gd_4_-TESMA and its parent peptide (VVGS-peptide) were determined in 50% (v/v) aqueous pooled serum from human male AB plasma, which was purchased from Sigma-Aldrich (Milan, Italy). Samples were dissolved in water (6.6 mM), diluted in serum (previously centrifuged at 8000 rpm for 15 min at 4 °C) at a final concentration of 0.6 mM (220 μL), and subsequently incubated at 37 °C. Aliquots (6 μL) taken after 0, 35, and 120 min were diluted in water at a final concentration of 0.06 mM. The samples were analyzed by RP-HPLC using an Atlantis dC18 column, 5 μm, 4.6 × 150 mm, as described in ref (47). The amount of intact compound was estimated by integrating the area under the corresponding elution peak monitored at 210 nm. Five replicate experiments for each compound were carried out. The KQLLWIRSGDRPWYYTS (HPLW peptide),^47^ unrelated to TESMA, was used as a positive control.

### Surface Plasmon Resonance Measurement of Binding Affinity

The binding affinity between tropoelastin (human recombinant tropoelastin, Advanced BioMatrix, Cat. No. 5052-1MG, Batch No. 7932) and Gd_4_-TESMA or Gd-TESMA was assessed by surface plasmon resonance (SPR) on a Biacore 2000 instrument (Cytiva). The instrument was equipped with a CM5 sensor chip carrying carboxymethylated dextran (Cytiva). First, the ligand was immobilized on the sensor chip surface by amine coupling: the flow cell was activated for 7 min by a 1-ethyl-3-(3-dimethylaminopropyl)carbodiimide hydrochloride (EDC)/*N*-hydroxysuccinimide (NHS) mixture. Then, tropoelastin was injected at 200 μM in a 10 mM sodium acetate buffer (pH 4) with a flow rate of 5 μL min^–1^ to reach an immobilization level corresponding to 3500–5000 resonance units (R.U.). The remaining active sites were blocked by 1 M of ethanolamine-HCl (pH 8.5) for 7 min. As a control for nonspecific interaction, another flow cell on the CM5 sensor chip was treated in the same manner but without adding tropoelastin. For the binding assay, the analyte was injected at 20 μL min^–1^ for 3 min at 25 °C and at different concentrations in the HEPES running buffer (pH 7.4, HBS-P, Cytiva) or 10 mM HEPES solution. This step was followed by a dissociation period of 2.5 min under the running buffer. The surface was regenerated by flowing 5 mM NaOH/1 M NaCl for 30–60 s at 30 μL min^–1^. A stabilization period of 4 min with the running buffer completed the cycle. The equilibrium dissociation constant (*K*_D_) and the kinetic association and dissociation rate constants (*k*_a_ and *k*_d_) were determined by curve-fitting using the 1:1 Langmuir binding model implemented in the Biaevaluation software 4.1.1.^48^ Before calculations, the nonspecific signal of the control channel was subtracted from the signal obtained from the functionalized channel.

### Murine Model of Atherosclerosis Progression

Male apolipoprotein E knockout (ApoE^–/–^) (*n* = 7) mice and wild-type C57BL/6J mice (*n* = 3) were purchased from Charles Rivers Laboratories (Edinburgh, UK). Eight-week-old ApoE^–/–^ mice were switched to a high-fat diet (HFD) containing 21% fat from lard and 0.15% (wt/wt) cholesterol (Special Diets Services, Witham, UK). ApoE^–/–^ mice were imaged at 16 weeks after HFD feeding. Age- and gender-matched wild-type mice were fed a normal chow diet for 16 weeks and were used as controls.

### *In Vivo* Imaging of Tropoelastin at 3 T

All imaging experiments were performed using a 3 T Philips Achieva MR scanner (Philips Healthcare, Best, the Netherlands) equipped with a clinical gradient system (30 mT m^–1^, 200 mT m^–1^ ms^–1^). Mice were imaged using a single-loop surface coil (diameter = 23 mm). Anesthesia was induced with 4–5% and maintained with 1–2% isoflurane during the MRI experiments. Anesthetized animals were placed in the prone position, and the brachiocephalic artery (BCA) was imaged. The BCA of atherosclerotic mice was scanned three times on 3 consecutive days. On day 1, mice were scanned up to 2 h after injection of Gd-ESMA (0.2 mmol kg^–1^). On day 2, the same animals were scanned up to 2 h postinjection of the monomeric tropoelastin-binding contrast agent Gd-TESMA (0.2 mmol kg^–1^). On day 3, the same mice were scanned up to 2 h postinjection of the tetrameric tropoelastin-binding contrast agent Gd_4_-TESMA (0.2 mmol kg^–1^). Twenty-four hours later, a subgroup of atherosclerotic mice (*n* = 3) was imaged up to 2 h after injection of the scrambled Gd_4_-TESMA (Gd_4_-_sc_TESMA; 0.2 mmol kg^–1^). Lastly, healthy control mice were scanned twice, on day 1 with Gd-ESMA and on day 3 with Gd_4_-TESMA, as described above. All injections were done intravenously.

### *In Vivo* MRI Protocol

Following a 3D GRE scout scan, contrast-enhanced angiography images were acquired for visualization of the aortic root, aortic arch, and brachiocephalic and carotid arteries with a FOV = 30 × 30 × 8 mm, matrix = 200 × 200, in-plane resolution = 0.15 × 0.15 mm (reconstructed = 0.10 × 0.10 mm), slice thickness = 0.5 mm, TR/TE = 15/6.1 (ms), and flip angle = 40°. The maximum intensity projection images were used to plan the subsequent late gadolinium enhanced (LGE) MRI. A 2D Look–Locker sequence planned perpendicular to the ascending aorta was used to determine the optimal inversion time (TI) for blood signal nulling with a FOV = 30 × 30 mm, matrix = 80 × 80, in-plane resolution = 0.38 × 0.38 mm, slice thickness = 2 mm, TR/TE = 19/8.6 (ms), TR between subsequent IR pulses = 1000 ms, and flip angle = 10°. Late gadolinium enhanced images were acquired with an inversion-recovery 3D fast gradient echo sequence every 30 min for up to 2 h postinjection of the contrast agent and were used to visualize and quantify contrast uptake. The FOV extended from the aortic root to the carotid arteries. Imaging parameters were FOV = 30 × 30 × 8 mm, matrix = 304 × 304, in-plane resolution = 0.1 × 0.1 mm, measured slice thickness = 0.5 mm, slices = 32, TR/TE = 28/8 (ms), TR between subsequent IR pulses = 1000 ms, and flip angle = 30°.

### MRI Image Analyses

The vessel wall area was calculated by manually segmenting the visually enhanced region of the vessel wall as seen on the LGE-MRI images using OsiriX (OsiriX Foundation, Geneva, Switzerland). To ensure that the segmented area encompassed the vessel wall, the LGE-MRI images were co-registered and fused with the magnetic resonance angiography images. The sum of LGE volume was calculated by adding the LGE area on each slice, encompassing the BCA, and multiplying by the slice thickness. The contrast-to-noise ratio (CNR) between the vessel and blood was calculated as CNR = (SI_wall_ – SI_blood_)/SD_noise_. SI = signal intensity and SD = standard deviation outside the body. The mean CNR in each animal is presented in the Results section.

### Histology

The aortic root, aortic arch, and brachiocephalic and carotid arteries of diseased and control mice were removed *en bloc*, pinned down to maintain the tissue morphology, and fixed in 10% formaldehyde for 48 h at 4 °C (*n* = 3). Tissues were embedded in paraffin and sectioned transversely (5 μm thick). Immunohistochemistry for tropoelastin was performed using an anti-mouse rabbit polyclonal antibody (1:100, Abcam, ab21600, Cambridge, MA, USA).

### Statistical Analysis

The relaxivities of the gadolinium complexes are expressed as mean ± SD. Multiple-group comparisons for repeated MRI measurements were performed using a Friedman test. MRI data are presented as median ± interquartile range (IQR). Statistical analysis was performed using PRISM (GraphPad Software, Inc., La Jolla, California). *P* values <0.05 were considered statistically significant.

## Supporting Information Available

Experimental design of the MRI study of Gd_4_-TESMA *in vivo*; HPLC traces, ESI+ MS, and NMR spectra of final products and reaction intermediates (PDF)

## Abbreviations

CNR: contrast-to-noise ratio
DIPEA: *N*,*N*-diisopropylethylamine
DOTA: 1,4,7,10-tetraazacyclododecane-1,4,7,10-tetraacetic acid
HBTU: *N*,*N*,*N*′,*N*′-tetramethyl-*O*-(1*H*-benzotriazol-1-yl)uronium hexafluorophosphate
HEPES: *N*-(2-hydroxyethyl)piperazine-*N*′-(2-ethanesulfonic acid)
IQR: interquartile range
LGE: late gadolinium enhancement
NMRD: nuclear magnetic resonance dispersion
PyBOP: benzotriazol-1-yloxytripyrrolidinophosphonium hexafluorophosphate
SPPS: solid phase peptide synthesis
SPR: surface plasmon resonance
TE: tropoelastin
