# Supplementary material for: Imaging of Dysfunctional Elastogenesis in Atherosclerosis Using an Improved Gadolinium-Based Tetrameric MRI Probe Targeted to Tropoelastin
Source: J Med Chem. 2021 Oct 18;64(20):15250–61. doi: 10.1021/acs.jmedchem.1c01286 (PMC8558862; doi:10.1021/acs.jmedchem.1c01286)
Supplement: Supplementary file 1 — jm1c01286_si_001.pdf [file jm1c01286_si_001.pdf]

Supporting Information

Imaging of Dysfunctional Elastogenesis in Atherosclerosis Using an Improved Gadolinium-Based Tetrameric MRI Probe Targeted to Tropoelastin

Federico Capuana,[1,§] Alkystis Phinikaridou,[2,§] Rachele Stefania,[1] Sergio Padovan,[3] Begoña Lavin,[2,4] Sara Lacerda,[5] Eyad Almouazen,[6] Yves Chevalier,[6] Laurence Heinrich-Balard,[7] René M. Botnar,[2,8] Silvio Aime,[9] and Giuseppe Digilio[10,*]

1 Department of Molecular Biotechnology and Health Sciences, University of Turin, Via Nizza 52, 10126 Turin, Italy.

2 School of Biomedical Engineering and Imaging Sciences, King’s College London, Westminster Bridge Road, SE1 7EH London, United Kingdom.

3 Institute for Biostructures and Bioimages (CNR) c/o Molecular Biotechnology Center, Via Nizza 52, 10126 Torino, Italy.

4 Department of Biochemistry and Molecular Biology, School of Chemistry, Complutense University, Ciudad Universitaria s/n, 28040 Madrid, Spain.

5 Centre de Biophysique Moléculaire, CNRS, UPR 4301, Université d’Orléans, Rue Charles Sadron, 45071 Orléans Cedex 2, France.

6 CNRS, LAGEPP UMR 5007, Univ Lyon, Université Claude Bernard Lyon 1, 43 boulevard du 11 novembre 1918, 69622 Villeurbanne, France.

7 INSA Lyon, CNRS, MATEIS, UMR5510, Univ Lyon, Université Claude Bernard Lyon 1, 69100 Villeurbanne, France.

8 Escuela de Ingeniería, Pontificia Universidad Católica de Chile, Avda.Vicuña Mackenna, 4860 Santiago, Chile.

9 IRCCS SDN, 80100 Napoli, Italy.

10 Department of Science and Technologic Innovation, Università del Piemonte Orientale “Amedeo Avogadro”, Viale T. Michel 11, 15121 Alessandria, Italy.

* Corresponding author: Giuseppe Digilio (giuseppe.digilio@uniupo.it)

§ These authors contributed equally

Table of Contents

Supporting Figure S1 ....S3

Supporting Figure S2 ....S4

Supporting Figure S3 ....S5

Supporting Figure S4 ....S6

Supporting Figure S5 ....S7

Supporting Figure S6 ....S8

Supporting Figure S7 ....S9

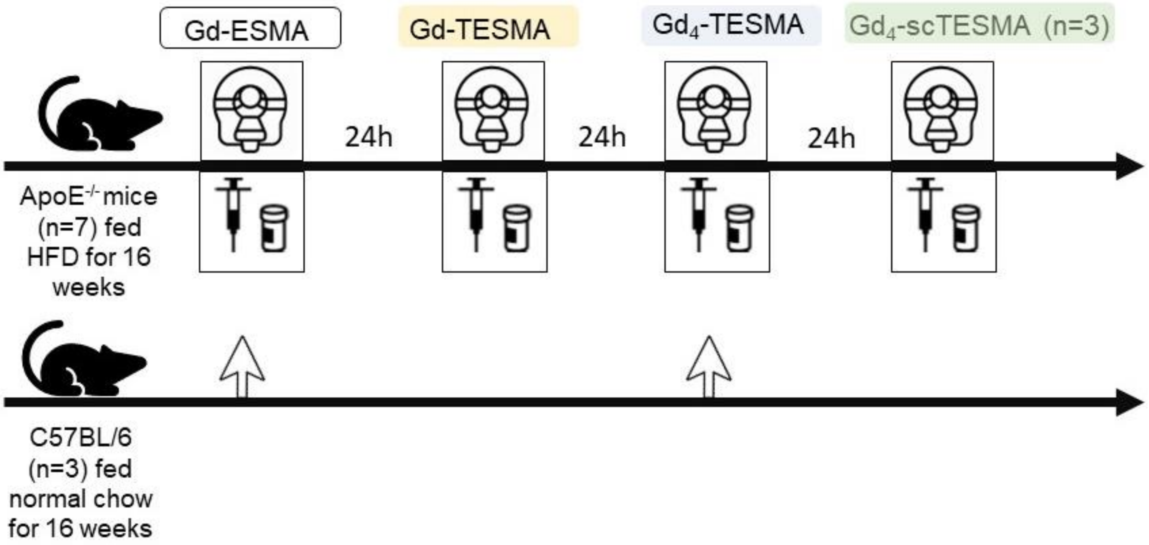

Figure S1. Experimental plan for MRI at 3T of atherosclerotic plaques in ApoE$^{-/-}$ mice.

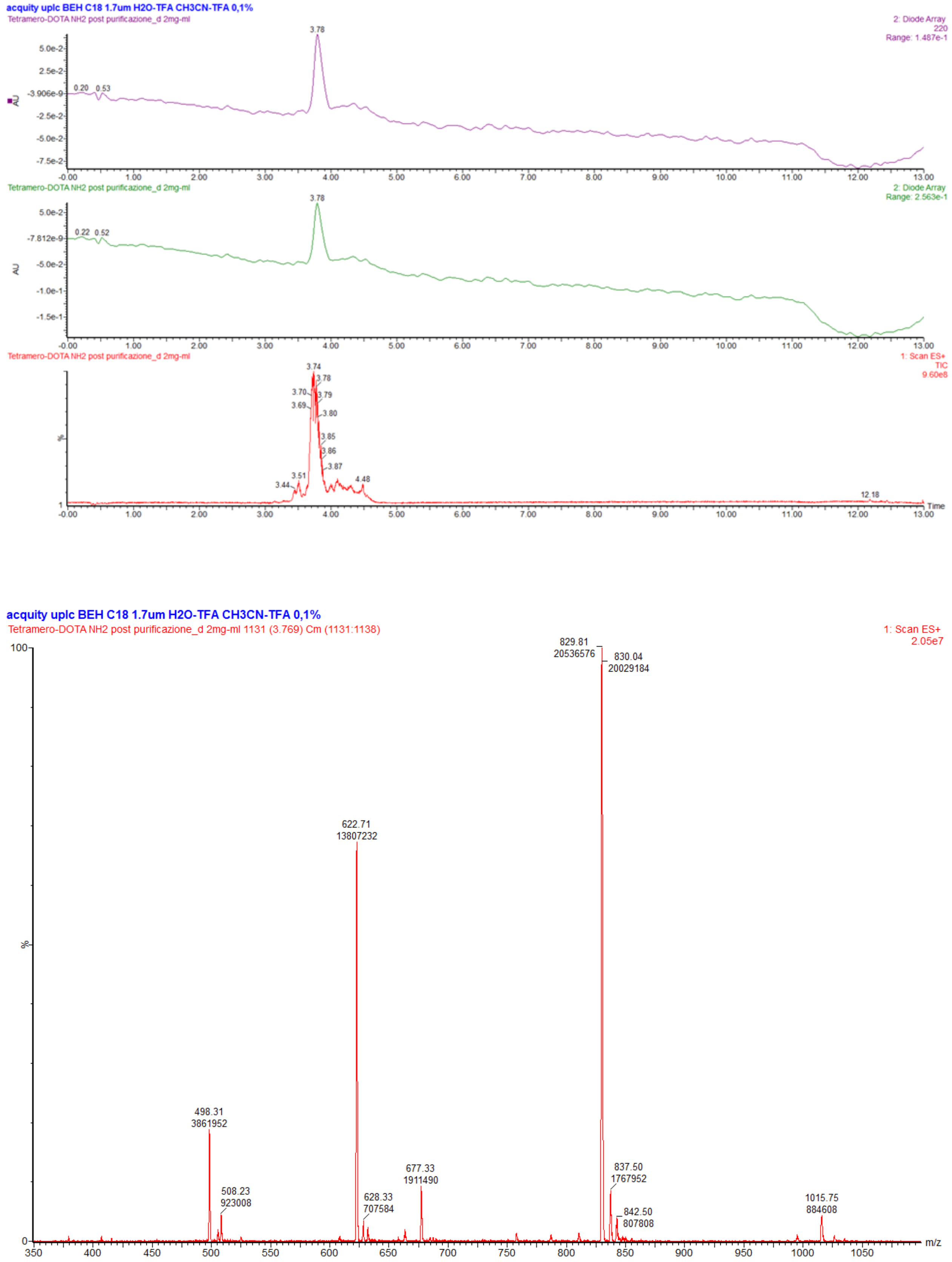

Figure S2. Top: UPLC chromatogram of $[DOTA]_4$-NH_2 (λ = 220 nm, diode array 200-400 nm, TIC scan ESI+). Retention time 3.7 min, purity of $[DOTA]_4$-NH_2 > 95%. Bottom: mass spectrum relative to the peak at 3.7 min. The peak was assigned to $[DOTA]_4$-NH_2 ($C_{110}H_{196}N_{28}O_{36}$): $[M+3H]^{3+}$ 829.8, $[M+4H]^{4+}$ 622.7, $[M+5H]^{5+}$ 498.3.

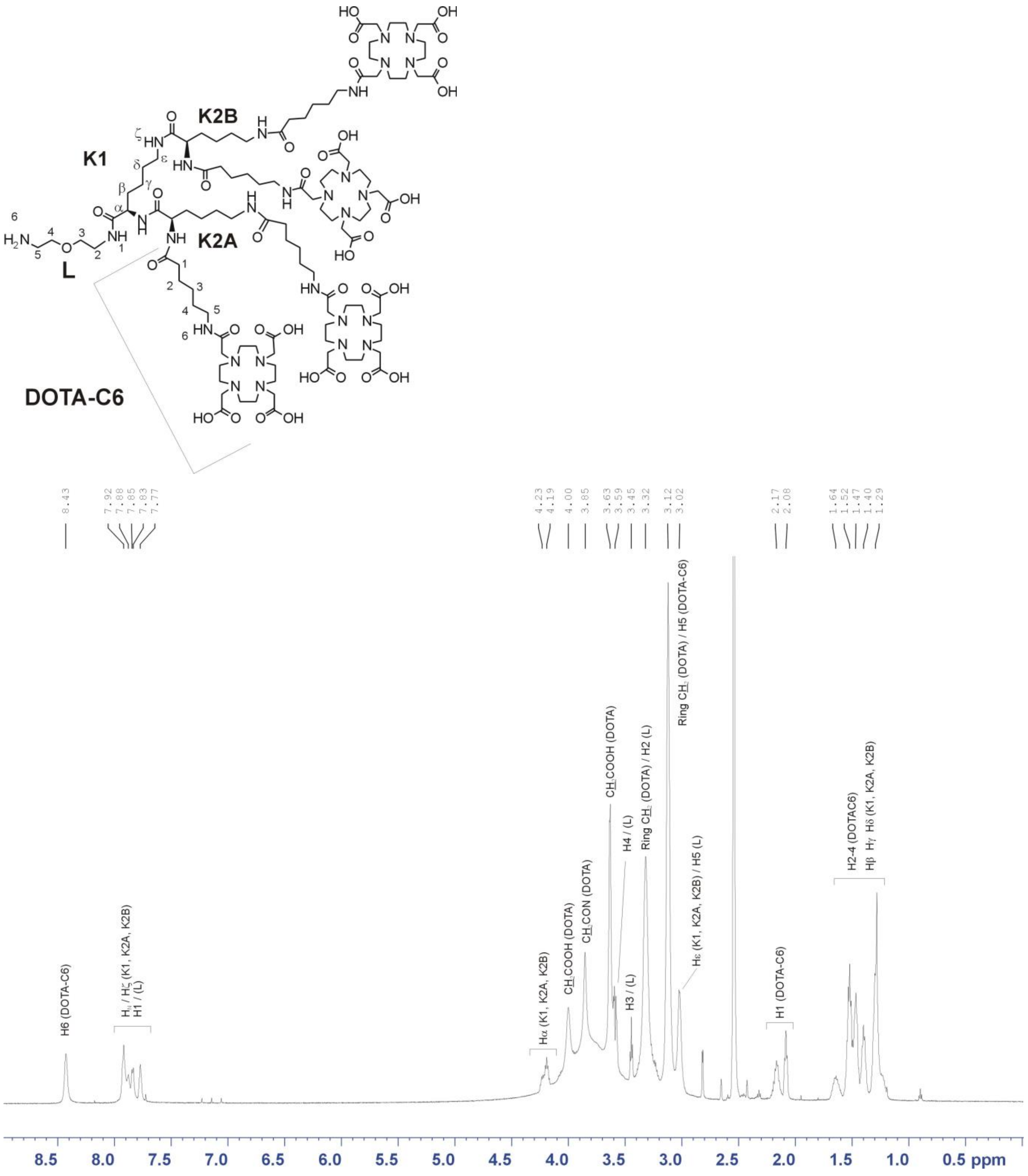

Figure S3. ^{1}H-NMR of $[DOTA]_4$-NH_2 (600 MHz, dmso-d_6, 37 °C) with signal assignment. Refer to the top right structure for signal labels. Signal assignment was achieved by 2D-TOCSY, 2D-NOESY and 2D-COSY NMR spectroscopy.

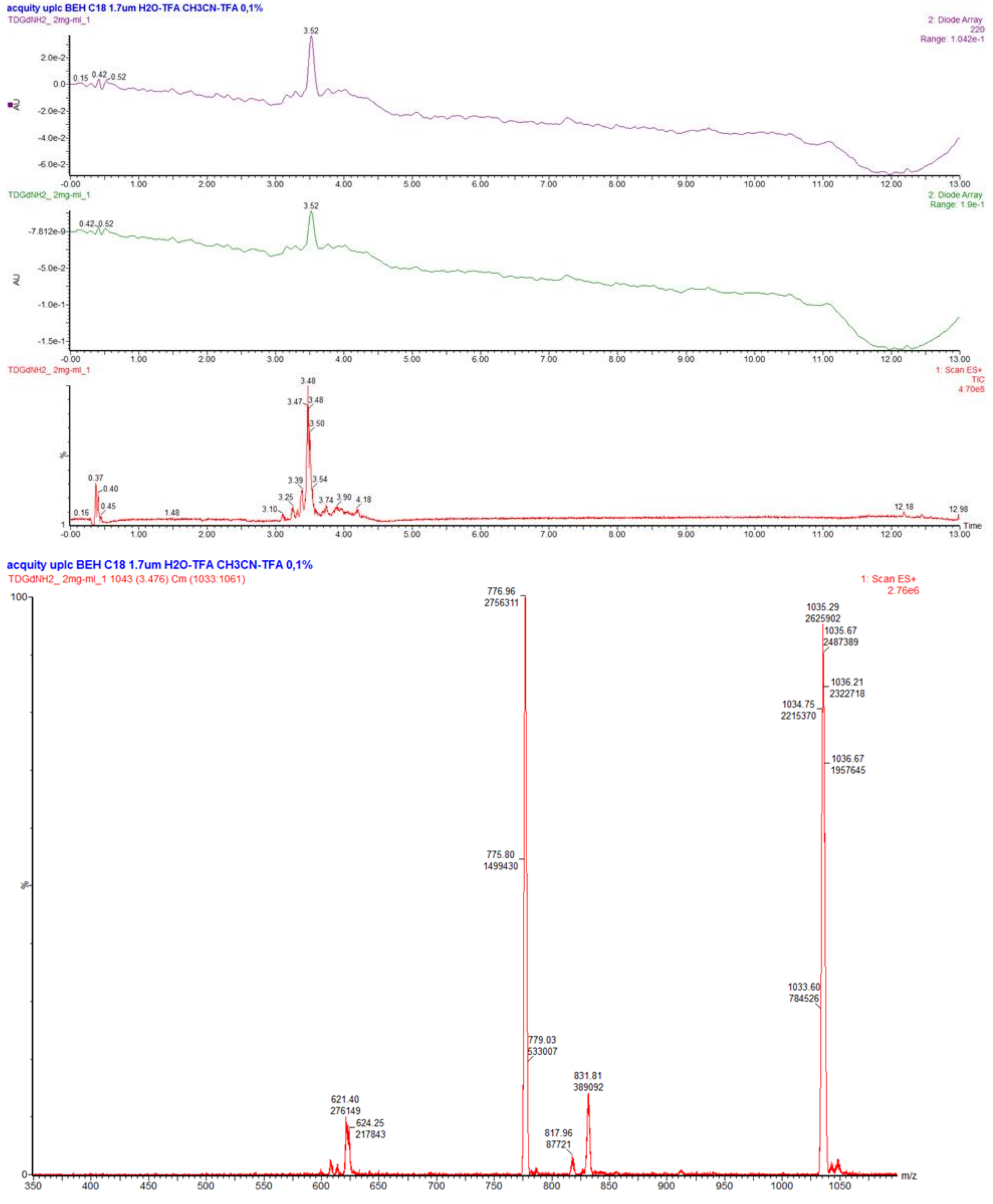

Figure S4. Top: UPLC chromatogram of [Gd-DOTA]$_4$-NH$_2$ (λ = 220 nm, diode array 200-400 nm, TIC scan ESI+). Retention time 3.5 min, purity of [Gd-DOTA]$_4$-NH$_2$ > 95%. Bottom: mass spectrum relative to the peak at 3.5 min. The peak was assigned to [Gd-DOTA]$_4$-NH$_2$ ($C_{110}H_{184}Gd_4N_{28}O_{36}$): $[M+3H]^{3+}$ 1035.3, $[M+4H]^{4+}$ 776.9.

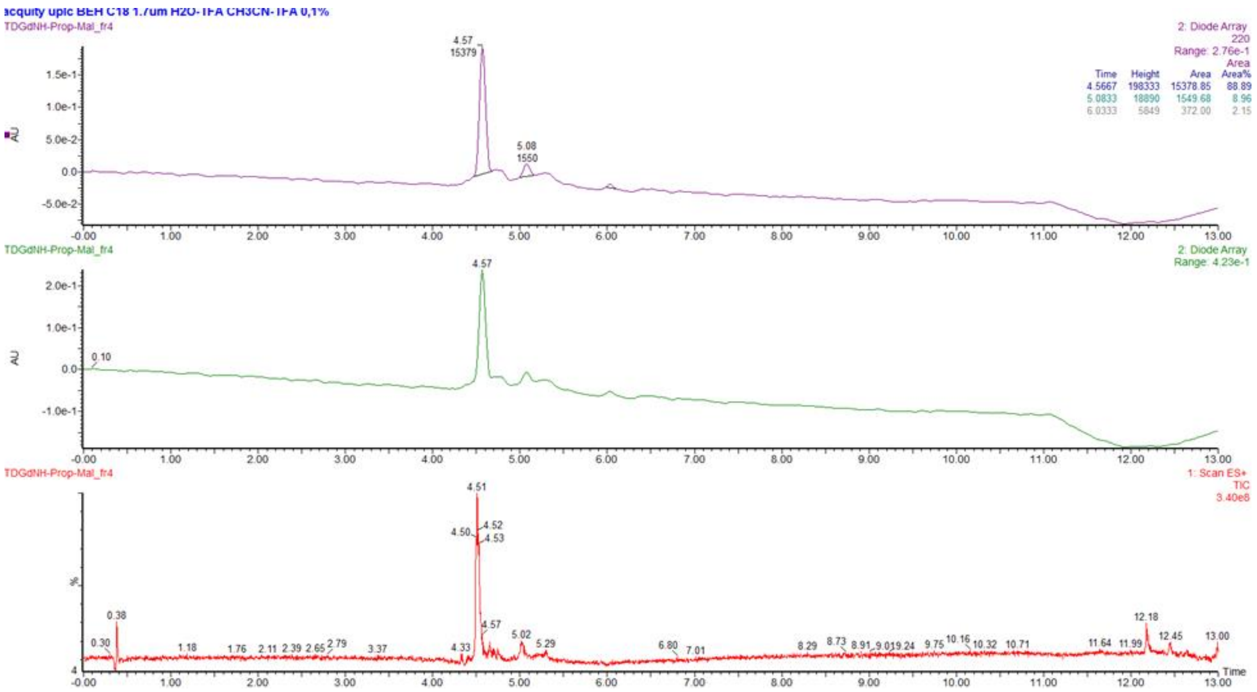

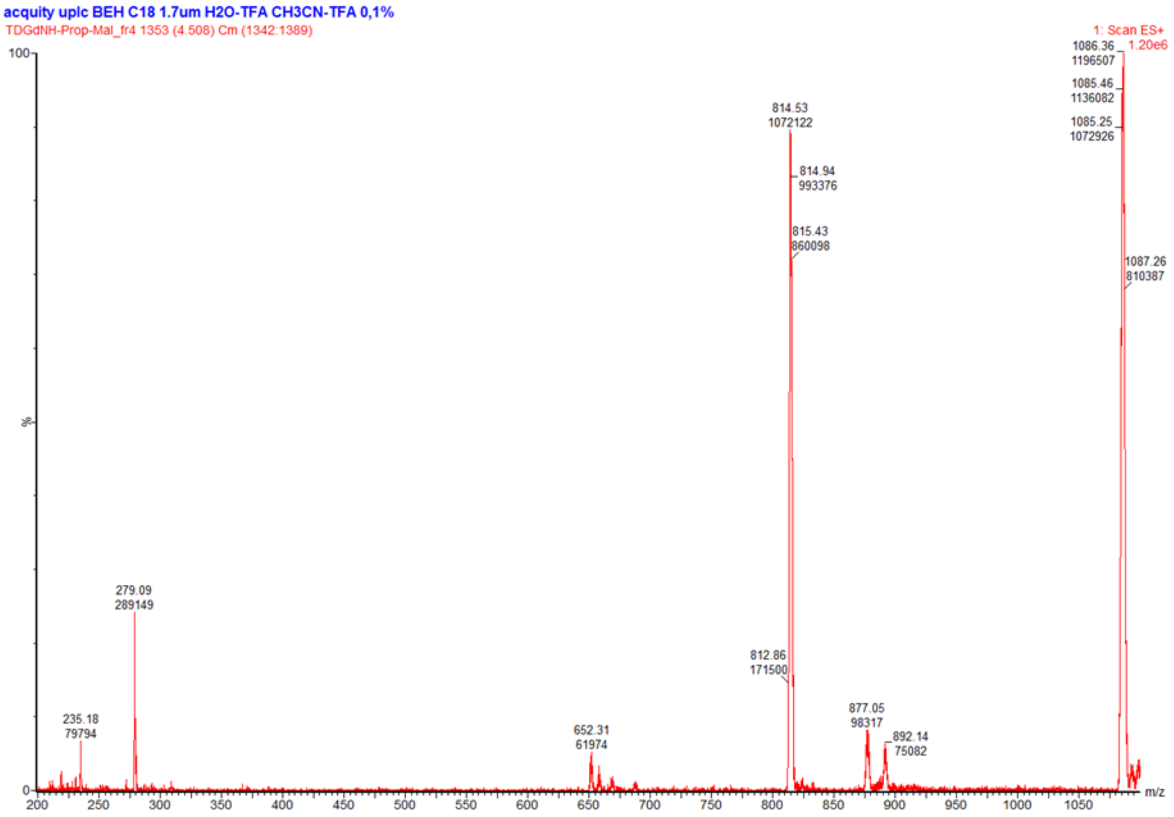

Figure S5. Top: UPLC chromatogram of [Gd-DOTA]$_4$-MI (λ = 220 nm, diode array 200-400 nm, TIC scan ESI+). Retention time 4.5 min, purity of [Gd-DOTA]4-MI > 88%. Bottom: mass spectrum relative to the peak at 4.5 min. The peak was assigned to [Gd-DOTA]$_4$-MI ($C_{117}H_{189}Gd_4N_{29}O_{39}$): $[M+3H]^{3+}$ 1086.3, $[M+4H]^{4+}$ 814.5.

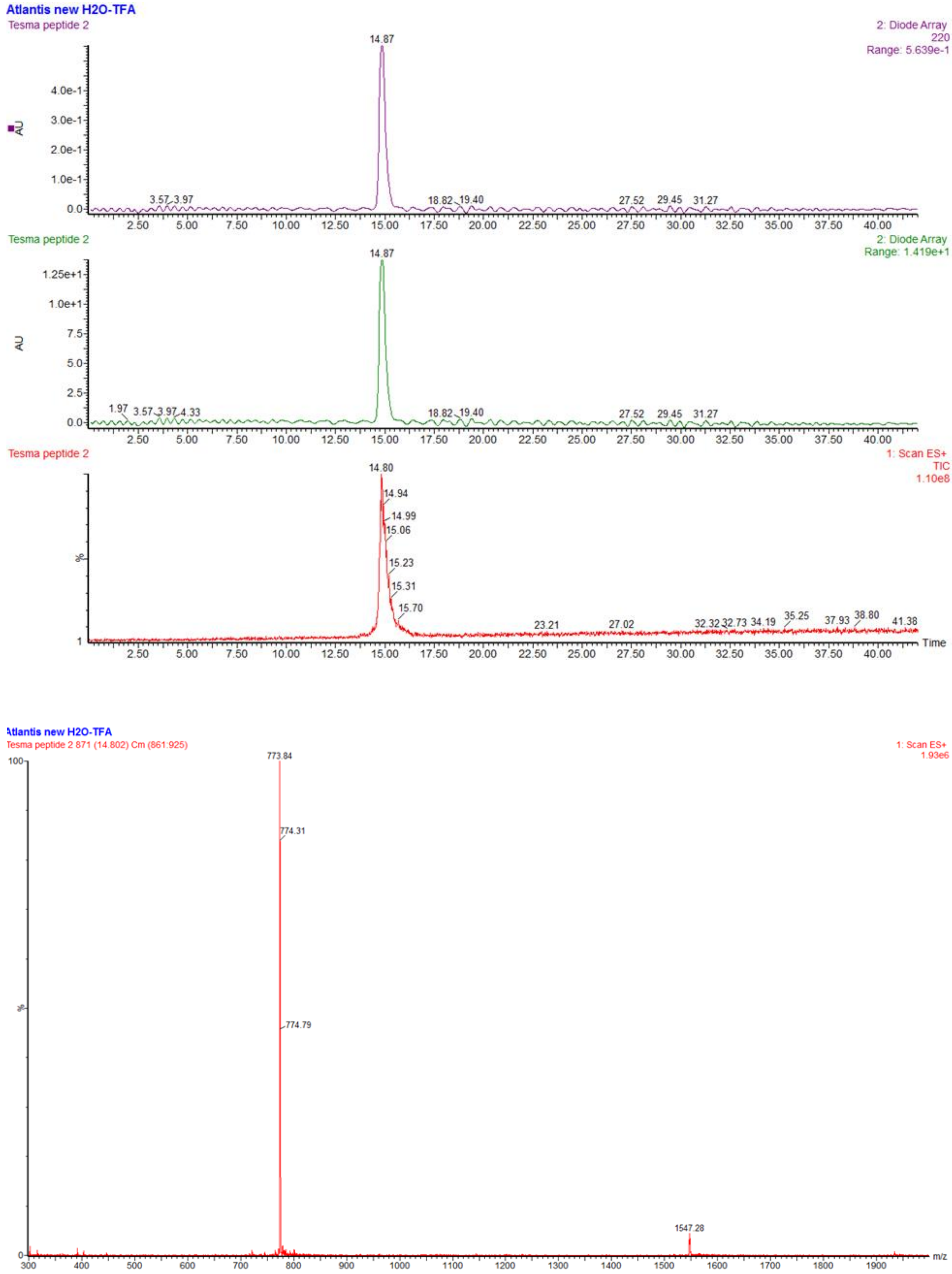

Figure S6. Top: HPLC-UV chromatogram of the C-VVGS peptide (λ = 220 nm, diode array 200-400 nm, TIC scan ESI+). Retention time 14.8 min, purity of C-VVGS peptide > 95%. Bottom: mass spectrum relative to the peak at 14.8 min. The peak was assigned to C-VVGS peptide ($C_{63}H_{103}N_{17}O_{26}S$): $[M+3H]^{3+}$ 1086.3, $[M+4H]^{4+}$ 814.5.

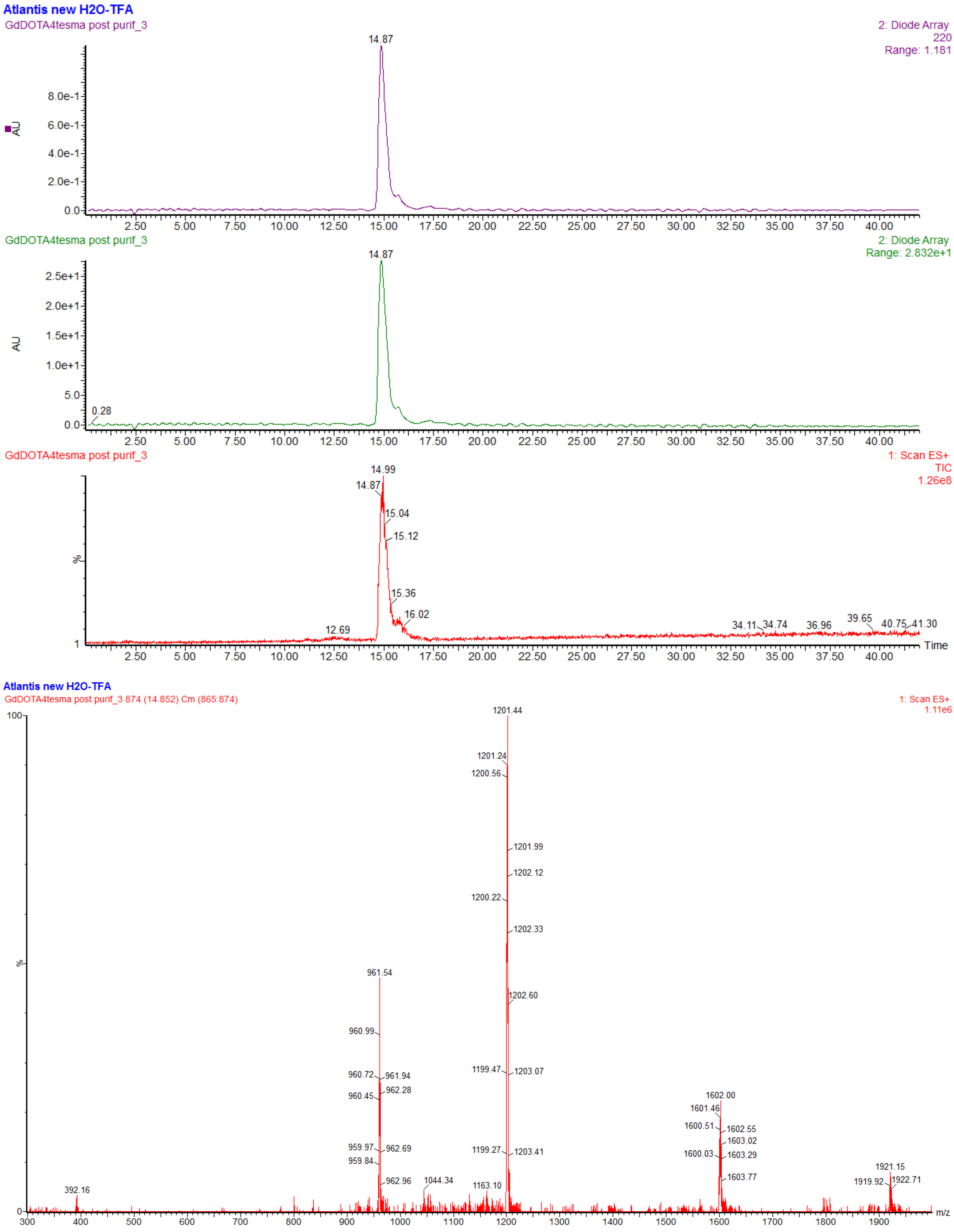

Figure S7. Top: HPLC-UV chromatogram of Gd_4-TESMA (λ = 220 nm, diode array 200-400 nm, TIC scan ESI+). Retention time 14.8 min, purity of C-VVGS peptide > 95%. Bottom: mass spectrum relative to the peak at 14.8 min. The peak was assigned to Gd_4-TESMA ($C_{180}H_{292}Gd_4N_{46}O_{65}S$): $[M+5H]^{5+}$ 962.1; $[M+4H]^{4+}$ 1201.4; $[M+3H]^{3+}$ 1602.0.
